# The Tip of the “Celiac Iceberg” in China: A Systematic Review and Meta-Analysis

**DOI:** 10.1371/journal.pone.0081151

**Published:** 2013-12-04

**Authors:** Juanli Yuan, Jinyan Gao, Xin Li, Fahui Liu, Cisca Wijmenga, Hongbing Chen, Luud J. W. J. Gilissen

**Affiliations:** 1 State Key Laboratory of Food Science and Technology, Nanchang University, Nanchang, Jiangxi, China; 2 College of Pharmaceutical Sciences, Nanchang University, Nanchang, Jiangxi, China; 3 School of Life Sciences and Food Engineering, Nanchang University, Nanchang, Jiangxi, China; 4 Department of Genetics, University Medical Centre Groningen, University of Groningen, Groningen, The Netherlands; 5 Sino-German Joint Research Institute, Nanchang University, Nanchang, Jiangxi, China; 6 Plant Research International, Wageningen University & Research Centre, Wageningen, The Netherlands; Charité University Medicine Berlin, Germany

## Abstract

**Objective:**

Until recently, celiac disease was considered to be rare in China. We aimed to estimate its true status.

**Methods:**

By searching the MEDLINE database and four Chinese full-text databases (CNKI, CBM, VIP and WANFANG) (up to August 2012), as well as two HLA allele frequency net databases and the Chinese Statistics Yearbook databases, we systematically reviewed the literature on definite and suspected cases of celiac disease, the predisposing HLA allele frequencies, and on gluten exposure in China. Meta-analysis was performed by analyzing DQ2, DQ8 and DQB1*0201 gene frequencies and heterogeneity in populations from different geographic regions and ethnicities in China.

**Results:**

At present, the number of reported celiac disease cases is extremely low in China. The frequencies of the HLA-DQ2.5 and HLA-DQ8 haplotypes were 3.4% (95% confidence interval 1.3–5.5%) and 2.1% (0.1–4.1%), respectively. HLA-DQ2 and HLA-DQ8 antigen frequencies were 18.4% (15.0–21.7%) and 8.0% (4.5–11.4%), respectively. The frequency of the DQB1*0201 allele was 10.5% (9.3–11.6%) and it was more common in the northern Chinese than in the southern Chinese populations. The chance of being exposed to gluten is rapidly increasing all over China nowadays.

**Conclusion:**

The data on HLA haplotyping, in conjunction with increasing wheat consumption, strongly suggests that the occurrence of celiac disease is more common in China than currently reported. Coordinated measures by the Chinese government, medical and agricultural research institutions, and food industries, would be justified to create more awareness about celiac disease and to prevent it becoming a medical and societal burden.

## Introduction

Celiac disease (CD) is an autoimmune disorder elicited in genetically predisposed individuals by the consumption of foods containing gluten-containing grains (i.e., wheat, barley, and rye) or ingredients made thereof. Gluten proteins, as a stimulator, activate the innate and adaptive immune responses in the intestinal mucosa, resulting in changes in the intestinal epithelial and lamina propria through infiltration of lymphocytes and mononuclear cells, crypt hyperplasia, and villous atrophy [1]. The gluten-induced mucosal damage classically involves the proximal part of the small intestine, with a huge loss of mucosal surface area and consequent malabsorption of nutrients, vitamins and minerals, which contributes to a wide variety of clinical symptoms. The clinical presentation of CD varies markedly with the age of the patient. The classical symptoms of CD in children are mostly gastrointestinal manifestations, including chronic diarrhea, steatorrhea, abdominal bloating and pain, malnutrition, and weight loss. In adolescents and adults, CD is more often diagnosed with non-specific abdominal discomfort or extra-intestinal symptoms (e.g., anemia, peripheral neuropathy, fatigue, reduced bone density). Moreover, some patients have no symptoms at all despite the presence of characteristic intestinal lesions, while various atypical clinical manifestaions and asymptomatic forms of CD mean that most cases remain un- or misdiagnosed. This has led to the concept of a “celiac disease iceberg”, with only the “tip” of the cases being seen. Furthermore, undiagnosed CD patients cannot receive timely treatment, and will have an elevated risk for developing secondary autoimmune disorders (type 1 diabetes, thyroid dysfunction, Addison's disease, etc.) as well as malignant cancer [1], [2].

CD is an intestinal disorder with a multi-factorial etiology. HLA and non-HLA genes combined with gluten consumption and other, less well-defined, environmental factors are involved in its development [3], [4]. It has been demonstrated that genetic predisposition to CD is specifically related to the prevalence of the human leukocyte antigen (HLA)-DQ2 and HLA-DQ8 heterodimers: carriers of these have an up to 13 times increased risk to develop CD [5]. Approximately 95% of CD patients inherit the HLA-DQ2.5 heterodimer (DQA1*0501-DQB1*0201) either in cis or in trans, and the others usually express the HLA-DQ8 heterodimer (DQA1*0301-DQB1*0302) [5]–[7]. Moreover, the gene dose of the number of DQB1*0201 alleles, but not of the DQA1*0501 alleles, is associated with a more severe form of CD, as assessed by villous atrophy at the time of diagnosis and after one year on a gluten-free diet (GFD) [8].

CD has recently been recognized as a common disease in most parts of the world. The prevalence in Caucasian populations is estimated to be at least 1% [2]. In some countries, such as Finland and Sweden, and in the Sahara, much higher frequencies of up to 5% have been reported [9]–[11]. In recent years, an increasing number of CD patients have been identified in the Middle East, India and North Africa, where it was historically considered to be an extremely rare disease [12]. Other countries have no or only inconclusive data on the prevalence of CD, e.g. the Asia-Pacific region, which suggests that CD is rare in China [13]. However, there is little known about the status of CD in China and an unseen “celiac iceberg”, with the prevalence of CD in adults being higher than previously suggested, cannot be excluded [14].

To estimate the current status of CD, as a landmark reference point, we performed a systematic review and meta-analysis based on Chinese and international literature reporting definite or suspected CD cases, the frequency of occurrence of the major predisposing genes (HLA-DQ2, HLA-DQ8 and HLA-DQB1*0201) in various populations, and the major environmental factor, i.e. dietary wheat and gluten consumption.

## Methods

### Search strategy

The literature retrievals were run on the Medline database (up to August 2012) and Chinese full-text databases CNKI (1979-Aug. 2012), CBM (1978-Aug. 2012), VIP (1989-Aug. 2012), and WANFANG (1982-Aug. 2012). Moreover, for the HLA genes, the Allele Frequency Net Database (http://www.allelefrequencies.net) and the Anthropology/Allele Frequencies of IHWG Projects in the dbMHC Database (http://www.ncbi.nlm.nih.gov/gv/mhc/) were applied. Data on differences in dietary habits in southern and northern Chinese populations, especially with regard to their consumption of wheat and rice as staple foods, were obtained from the Chinese Statistics Yearbook databases.

In searching for clinical cases, our subject terms included “celiac disease”, “celiac sprue”, “non-tropical sprue”, “gluten intolerance”, and “gluten-sensitive enteropathy”. In searching for the predisposing gene frequency, the strategy subject terms were: “HLA antigens”, “histocompatibility antigens class II” or “major histocompatibility complex”, and the text terms were: “DQ2”, “DQw2”, “DQ8”, “DQw8”, “DQB1”, or “DQβ1”. Studies on dietary habits were identified with the following combinations of search text terms: “wheat”, “wheat flour”, “rice” “gluten”, “gliadin”, “bread”, “steamed bread”, “noodle”, “instant noodle”, “biscuit”, “cake”, “steamed stuffed bun”, “dumpling” and “consumption”, “purchase”, “ingestion”, “intake”. Searches in Medline/PubMed databases also included “China” or “Chinese” in the text, in addition to the above terms. References cited in the original articles and reviews we identified were curated manually. No restrictions were applied during the search on the publication time, status, or language of publication.

### Data abstraction

Data were abstracted by using a predetermined electronic form by one reviewer, and verified by a second reviewer. Discrepancies in data extraction were resolved by consensus. In order to ensure the quality of the literature in Chinese, we only selected those papers published in key Chinese journals which were included in *A Guide to the Core Journals of China* (Beijing: Peking University Press) [15]. This guide is widely used and accepted by Chinese academia. For the consumption of rice and wheat in China, we collected relevant statistics from national and provincial yearbooks.

Publications on gene frequencies needed to meet the following criteria: (1) subjects in the tests had to be representative of the general healthy population, unrelated, and include both males and females; and (2) the allele frequency must have been determined by a validated calculation method. In order to analyze and compare data, the HLA DQB1*0201 and DQB1*02 allele frequencies (AF) were estimated by direct counting, using the unified formula: AF  =  (sum of each individual allele)/2n, where n =  number of subjects. If a study was reported in more than one publication, only the most recent and complete information was included in the meta-analysis.

Data on gene frequencies were obtained from two kinds of Chinese studies: on the polymorphism of HLA in the healthy population, and on HLA associations with various specific diseases (e.g. essential hypertension, type 1 diabetes). To avoid a biased estimation of the gene frequency in a community, we excluded studies in which the subjects were only male or female. From the HLA association studies, we only considered the data from the healthy control group.

### Data synthesis and meta-analysis

Data on gene frequencies were divided into four categories of frequencies for: (1) HLA-DQ haplotypes: HLA-DQ2.5 (DQA1*0501-DQB1*0201) and HLA-DQ8 (DQA1*0301-DQB1*0302); (2) HLA-DQ2 and HLA-DQ8 antigens; (3) DQB1*0201 alleles; and (4) DQB1*0201/02 alleles. (HLA-DQ2 antigens refer to cell surface receptor-type proteins, which are a combination of two alleles: an HLA-DQB1 allele (which can be either the DQB1*0201 or the DQB1*0202 allele) and an HLA-DQA1 allele (which is the DQA1*0501 allele). Similarly, HLA-DQ8 consists of the DQB1*0302 and DQA1*0301 alleles.) Because Han, other ethnic minorities, and northern and southern subgroups may have different genetic backgrounds, the data were further subdivided by taking the nationality and place of residence of subjects into account.

The Yangtze River appeared to be a genetic demarcation line between southern and northern Mongoloids in China, as shown by multi-locus data (130 alleles at 38 loci) [16], [17] and the spatial genetic structure of three loci (ABO, HLA-A and TPOX) using the contour area multi-fractal model (CAMM) [18]. In accordance with Du et al. [17], the five provinces across the Yangtze River, along with Hubei, Sichuan and Yunnan were denoted as southern China, whereas the provinces Anhui and Jiangsu were denoted as part of northern China.

Data were analyzed using the statistical software package STATA 11 (Stata Corp, College Station, Tx, USA). The results were presented using Forest plots. The exact 95% confidence intervals (CI) were computed using the Clopper-Pearson method. Heterogeneity between the studies was assessed by chi-squared (chi2) and I-squared (I2) tests. Pooled effect size and 95% confidence interval were estimated using a random-effect model if there was marked heterogeneity (I2>50%) and by a fixed effect model in the other cases. The Z test was used to estimate the statistical significance of the effect of data pooling, which appeared valid at p<.05.

In order to analyze the differences in DQB1*0201 allele frequency between southern and northern populations, and Han and ethnic minorities, we analyzed the frequency of the heterogeneity between different subgroups. Heterogeneity was considered significant and highly significant when p values differed at the <0.05 and <0.01 level, respectively. If there was marked evidence of heterogeneity between studies in these subgroups, additional analyses of the subgroups were performed using the following criteria: those reports in which subjects and their families had lived in the same geographic area for at least three generations, those reports with ≥100 subjects, and those reports in which subgroups were identified according to differences in PCR-based DNA typing analysis.

In addition, we drew a DQB1*0201 and DQB1*0201/02 allele geographic distribution map for China. If two or more studies had been performed on the DQB1*0201 allele frequency of populations with the same place of residence, the weighted averages of gene frequencies were calculated. Moreover, when both DQB1*0201 and DQB1*0201/02 allele frequencies of individuals were available from the same province, only the data of the DQB1*0201 allele was included in our analysis.

## Results

### Definite and suspected cases of CD

**Figure 1:**
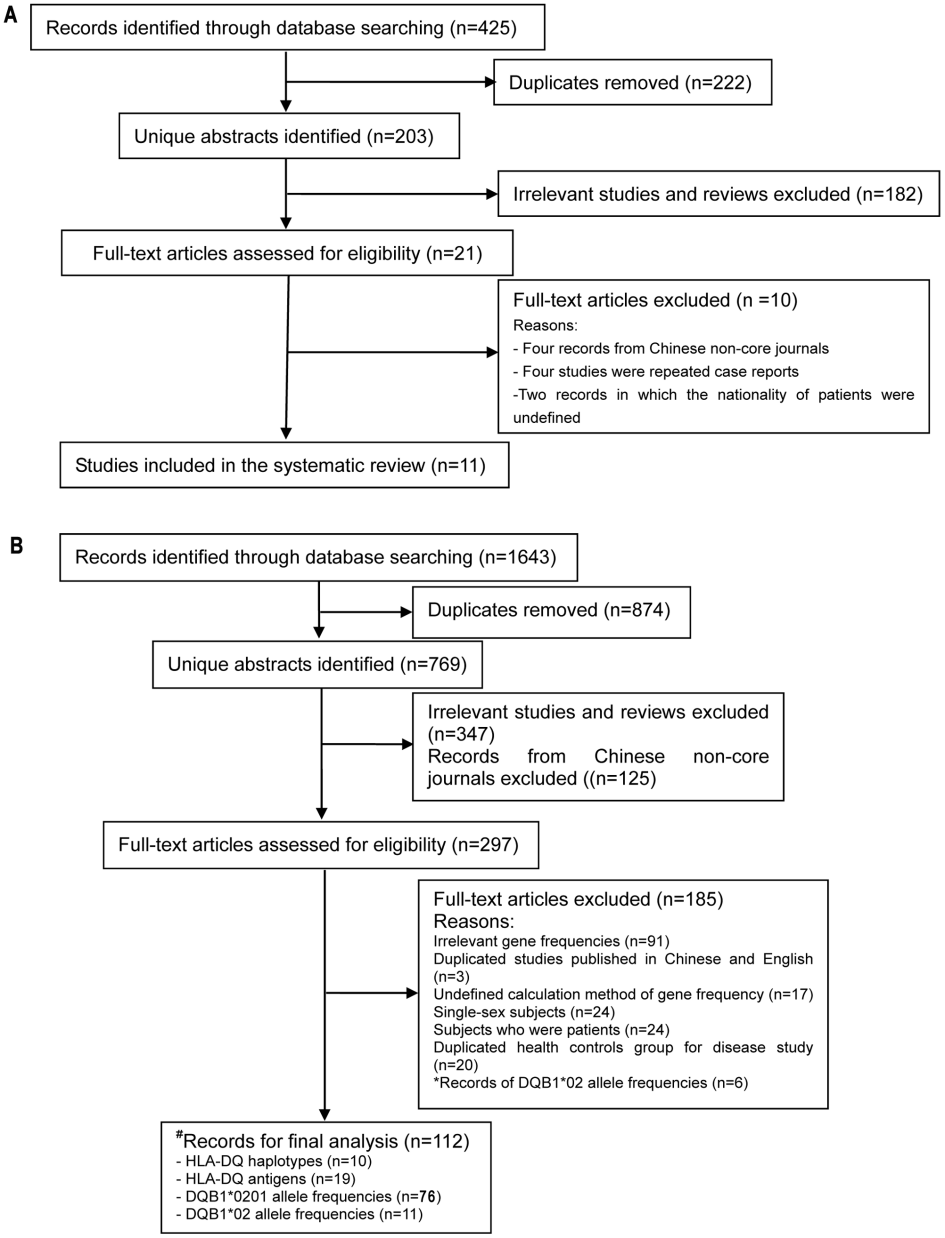
Flow diagram for search results. A: Flow chart for search results of celiac disease cases in the Chinese population. B: Flow chart for search results of CD-predisposing gene frequencies in the Chinese population. n, number of records; *In cases with both DQB1*0201 and DQB1*0201/02 allele frequencies of individuals from the same province, only the data of the DQB1*0201 allele were included in this research; ^#^Four records included data on haplotypes and DQB1*0201 or DQB1*0201/02.

We identified 425 potentially relevant studies, of which 11 met our inclusion criteria for the systematic review (Figure 1A). These studies reported 22 CD cases confirmed by duodenal biopsy and GFD [19]–[24], and four CD cases were diagnosed through serologic testing and GFD [25]–[27] (Table 1). Two of the 26 cases were refractory CD (RCD) [21], [27]. Moreover, there were seven CD cases reported among 2,400 Chinese patients with gastrointestinal disease who had had an endoscopy using an OMOM capsule (a wireless capsule endoscopy system, developed by Jianshan Science and Technology Ltd., Chongqing, China) in 2010 [28]. Another study showed significantly higher levels of IgA EMA and IgG EMA in 136 patients with symptoms of CD than in 50 control individuals [14].

**Table 1:** Characteristics of included studies about CD cases diagnosed by biopsy and a GFD.

First author, Year	Article type	The number of suspected CD patients, (gender, age)	Ethnic group/Region	Clinical presentations	Diagnostic method	Treatment	Diagnosis
Freeman HJ 2003 [19]	Original paper	1 female, 69 years	NA/Northern	Abdominal pain, 10 kg weight loss, Iron deficiency anemia, folate and B_12_ deficiency, hypomagenesemia	Small intestinal biopsy: Crypt hyperplastic, villous atrophy	GFD for at least 3 months: Normalization of the abnormal small intestinal structural changes	CD
Liu ZJ 2007 [20]	Case report	1 male, 76 years	NA	Chronic diarrhea for 5 years, 15 kg weight loss, chronic nonspecific abdominal complaints associated with elevated serum IgA and IgG, hypergammaglobulinemia, and persistent increased serum amylase.	HLA typing: HLA–DQ2; Serology testing: IgA AGA(+), IgG AGA(+), IgA EMA(+), IgG EMA(+), anti-nuclear antibodies(+); Duodenal biopsies: crypt hyperplasia, partial or total atrophy, and intraepithelial lymphocytosis	GFD for 6 months: Remained healthy, with no clinical, laboratory or imaging evidence of disease.	CD
Lok KH 2008 [21]	Case report	1 female, 52 years	NA	Chronic diarrhea and abdominal pain for more than 6 months, 14 kg weight loss, generalized edema, malnutrition	HLA typing: HLA–DQ8; Serology testing: IgA AGA(+), IgA EMA(+); Duodenal biopsies: crypt hyperplasia, partial or total atrophy, intraepithelial lymphocytosis	GFD for 3 months: severe diarrhea, nutritional status deteriorated. Corticosteroid therapy: symptoms subsided, nutritional status improved	RCD
Jiang LL 2009 [22]	Original paper	4 (3 males, 1 female), 28–73 years	Han/Zhejiang	Chronic diarrhea, 8–15 kg weight loss, debilitation, anemia, decreased levels of globulin, albumin and total cholesterol, and electrolyte imbalance	Duodenal biopsies: Blunting of the villi, crypt hyperplasia, a large number of lymphocytes infiltrating the epithelial cells	GFD for 2 months: Symptoms disappeared, 5–8 kg weight gaining	CD
Xiu LB 2010 [23]	Case report	1 male, 21 years	NA	Recurring diarrhea for 19 years, malnutrition, growth retardation	Serology testing: IgA EMA(−) Duodenal biopsy: villous atrophy, flat mucosa.	GFD for 6 weeks: diarrhea disappeared and body weight and growth increased.	CD
Wang XQ 2011 [24]	Invited review	14 (12 males, 2 females), 0.5–12 years	NA	Chronic diarrhea, failure to thrive, weight loss, weakness, dystrophy and anemia	Serology testing: IgA EMA (+) 9 cases; IgA EMA (−) 3 cases; IgA tTGs (+) 14 cases; Duodenal biopsy: pathological changes of mucosa: Marsh I: 1 case, Marsh II: 2 cases, Marsh III: 11 cases	GFD for one month: symptoms improved, diarrhea diminished	CD
Wu J 2010 [25]	Original paper	7 (3 males, 4 females), 20–64 years	Han/Jiangsu	Diarrhea-predominant irritable bowel syndrome: 6 cases,Insulin-dependent diabetes mellitus: 1 case	Serology testing: IgA tTGs (+) 1 case; IgG AGA (+) 5 cases; IgA tTGs (+) and IgG AGA (+): 1 cases.	2 out of these 7 patients followed a GFD for one year: diarrhea stopped	Suspected CD
Sun F 2008 [26]	Case report	1 female, 15 months	Han/NA	Diarrhea, poor weight gain, skin rash	Serology testing: IgA EMA(+), IgA tTGs (+), IgG tTGs (−),IgA AGA(−), IgG AGA(−), IgA ARA(−), IgG ARA(−), IgE antibodies to foods and inhalant allergens (−)	GFD for 1 month: diarrhea and rash disappeared, body weight increased	Suspected CD
Chen J 2012 [27]	Case report	1 male, 6 years	NA	Recurring diarrhea and vomit for 5 years, progressive weight loss, dental dysplasia and dental caries, malnutrition, vitamin deficiency, anemia	Serology testing: IgA EMA(+), IgA AGA (+)	GFD for 9 month: severe diarrhea, repeated vomiting, elevated IgA EMA and IgA AGA. Oral prednisone therapy: symptoms improved	RCD

+ positive; -negative

Abbreviations: NA, Not available in the content of the study; CD, celiac disease; RCD, refractory celiac disease; GFD, gluten-free diet;

IgA AGA: IgA anti-gliadin antibodies; IgG AGA: IgG anti-gliadin antibodies; IgA EMA: IgA anti-endomysial antibodies; IgG EMA: IgG anti-endomysial antibodies; IgA ARA: IgA anti-reticulin antibodies; IgG ARA: IgG anti-reticulin antibodies; IgA tTGs: IgA anti-tissue transglutaminase; IgG tTGs: IgG anti-tissue transglutaminase.

Three out of four original papers were on screening CD in at-risk groups in China, chronic diarrhea patients [22], [24], diarrhea-predominant irritable bowel syndrome, and insulin-dependent diabetes mellitus [25]. In the table, we only show the characteristics of suspected CD patients in these at-risk groups. Another original paper [19] was about CD in Asian-Canadian adults, one out of 14 adult CD cases was born in Northern China in 1924 and then emigrated from China to Canada in 1973. Sun F [26] reported two CD cases, but one case was of European descent.

### CD-predisposing gene frequencies

We identified 1,643 relevant studies through our searches, of which 112 were eligible for our final analysis (Figure 1B). The characteristics of the studies we included are shown in Tables S1, S2, S3, S4.

#### Frequencies of HLA-DQ haplotypes (HLA-DQ2.5, HLA-DQ8)

**Figure 2:**
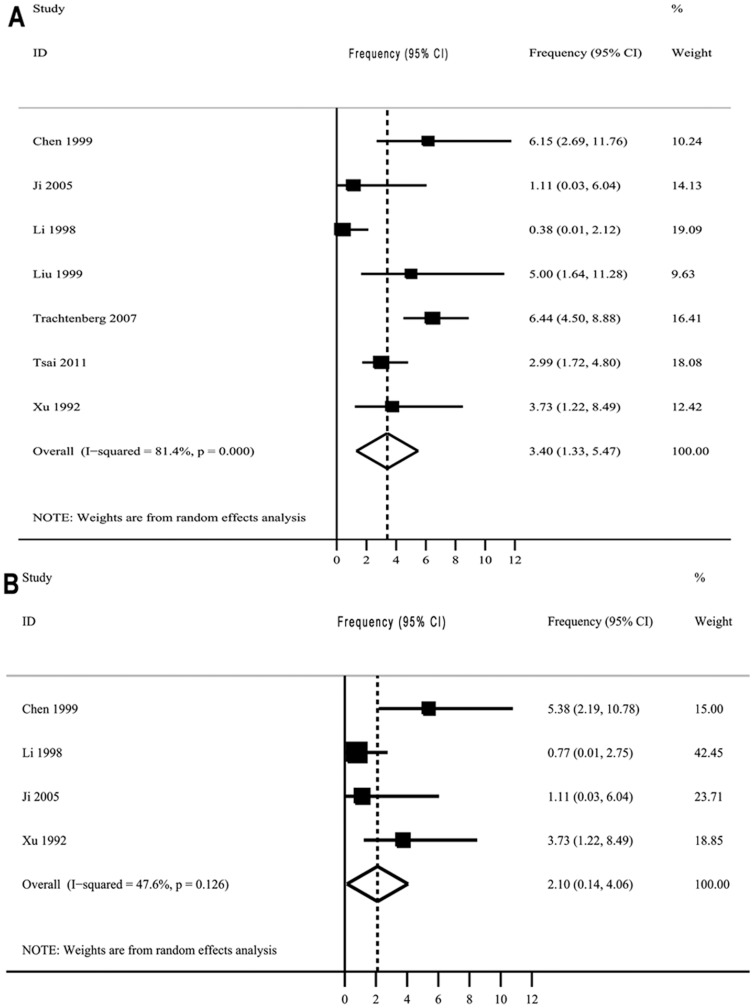
The frequencies and weightings of HLA-DQ2.5 and HLA-DQ8 haplotypes in the Chinese population. A: HLA-DQ2.5; B: HLA-DQ8. Abbreviations: CI, confidence interval. The data sources are given in Table S1.

Ten reports were found on the frequency of the HLA-DQ haplotypes (HLA-DQ2.5, HLA-DQ8) in the Chinese population (Table S1). The pooled frequencies of the DQA1*0501-DQB1*0201 and DQA1*0301-DQB1*0302 haplotype were 3.40% (95%CI, 1.33–5.47%) and 2.10% (95%CI, 0.14–4.06%), respectively (Figure 2). The cumulative numbers of individuals genotyped were 889 and 307, respectively. In addition, a survey in 160 unrelated, healthy Han individuals living in Jiansu Province showed frequencies of DQA1*0501-DQB1*0201/02 and DQA1*0301/02/03-DQB1*0302 haplotypes of 7.2% and 4.7%, respectively [29]. The frequencies of DQA1*05-DQB1*0201and DQA1*03-DQB1*0302 haplotypes in 226 Han individuals in Hunan province were 3.5% and 3.8%, respectively [30]. A survey in 476 Chinese individuals showed a frequency of DQA1*05-DQB1*0201 of 3.3% [31].

#### Frequencies of HLA-DQ2 and HLA-DQ8 antigens

**Figure 3:**
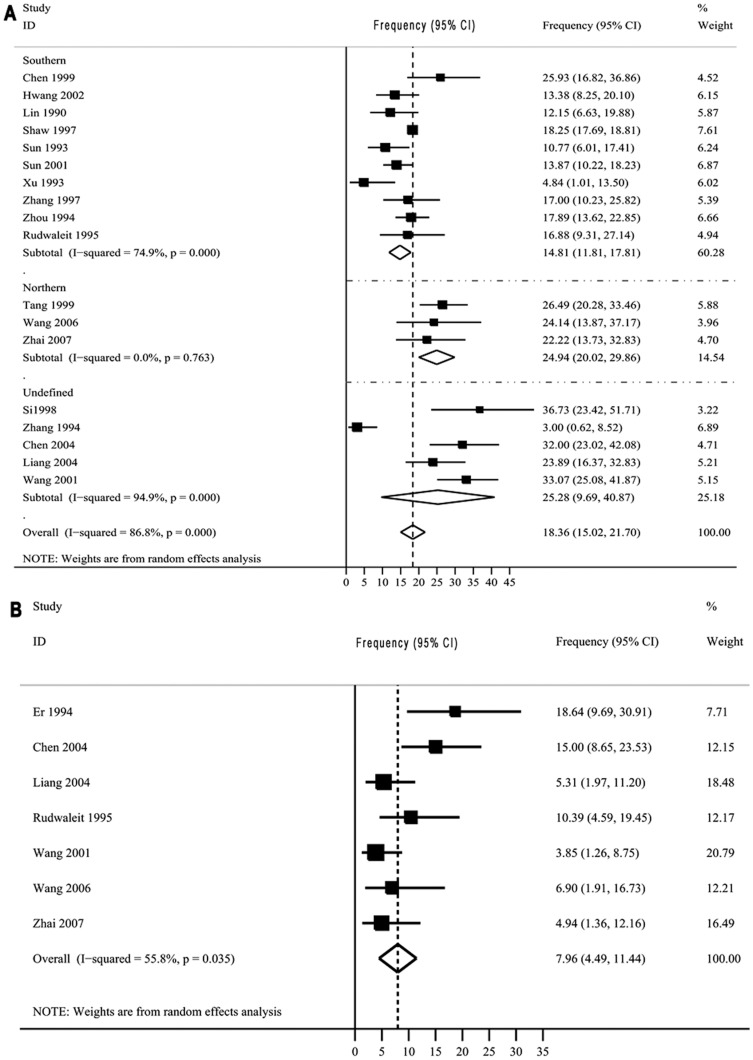
The frequencies and weightings of HLA- DQ2 and HLA-DQ8 antigens in the Chinese population. A: HLA-DQ2; B: HLA-DQ8. Antigen frequency  =  sum of each individual antigen/n, where n  =  total number of subjects. Abbreviations: CI, confidence interval. The data sources are given in Table S2.

In 19 studies, a standard microdrop lymphocyte cytotoxicity test and a PCR-SSP (polymerase chain reaction-sequence specific primers) test were used to analyze the occurrence of HLA-DQ antigen in the Chinese population (Table S2). The pooled frequencies of the HLA-DQ2 and HLA-DQ8 antigens were 18.36% (95%CI, 15.02–21.70%) and 7.96% (95%CI, 4.49–11.44%), respectively. The cumulative numbers of individuals tested were 20,316 and 618, respectively. The HLA-DQ2 antigen was more common in the northern subgroup (24.94%, 95%CI, 20.02–29.86%) than in the southern subgroup (14.81%, 95%CI, 11.81–17.81%) (Figure 3).

#### DQB1*0201 allele frequency in the Chinese

Data on DQB1*0201 allele frequencies in China were extracted from 76 articles (Table S3).

**Figure 4:**
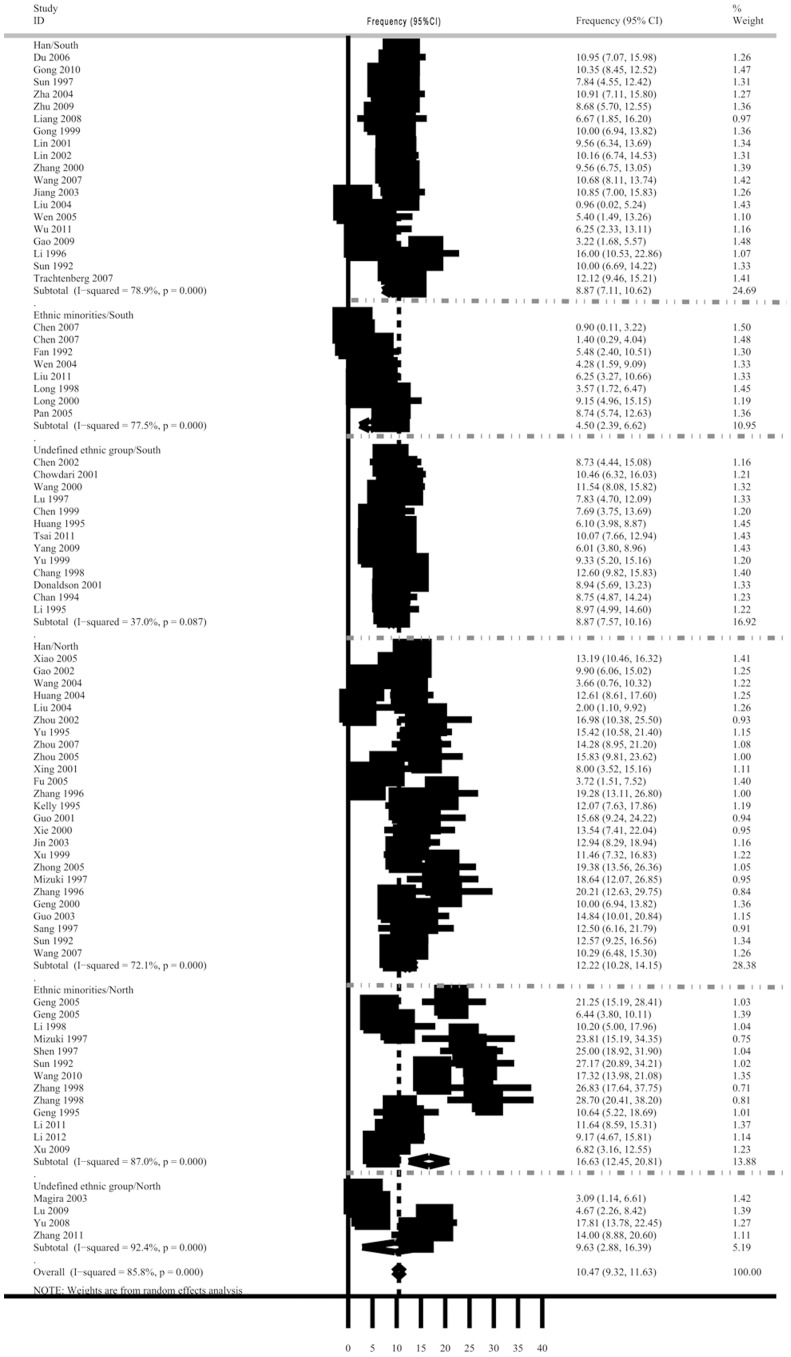
The DQB1*0201 allele frequency and its weighting in Chinese populations. Abbreviations: CI, confidence interval. The data sources are given in Table S3.

The overall DQB1*0201 allele frequency was 10.47% (95%CI, 9.32–11.63%) in the Chinese population, and the cumulative number of individuals tested was 9,095. In the northern population, the frequency was 13.27% (95%CI, 11.38–15.15%), and in the southern population it was 8.02% (95%CI, 6.74–9.30%) (Figure 4).

Taking into account the different genetic backgrounds among the Chinese population, we also analyzed the DQB1*0201 allele frequency in southern and northern Han groups and in the ethnic minorities. The DQB1*0201 allele frequencies in the Han populations and ethnic minorities were 9.31% (95%CI, 8.31–9.92%) and 6.07% (95%CI, 5.30–6.84%), respectively. The DQB1*0201 allele was more common among the northern ethnic minorities (16.63%, 95%CI, 12.45–20.81%) and northern Han groups (12.22%, 95%CI, 10.28–14.15%) than in the southern ethnic minorities (4.50%, 95%CI, 2.39–6.62%) and southern Han groups (8.87%, 95%CI, 7.11–10.62%). The differences between the northern and southern ethnic minorities were significant (Figure 4).

#### Additional subgroup analyses

The following subgroups were also analyzed separately: subjects and their families who had lived in the same geographic area for at least three generations, groups of ≥100 subjects, and four subgroups (PCR-SSO, PCR-RFLP, PCR-SSP and PCR-SBT) which showed differences in their PCR-based DNA typing analyses. All the results remained consistent with the overall analysis of all the groups, and the major subgroup analyses showed that the DQB1*0201 allele was more common or similar in the Han groups and ethnic minorities. However, our analysis of those studies which had adopted a PCR-SSO typing method showed a difference between the Han groups (12.57%, 95%CI, 11.22–13.91%) and the ethnic minorities (15.70%, 95%CI, 8.09–23.31%) (Table S5).

#### The geographic distribution of the DQB1*0201 allele in Chinese population groups

**Figure 5:**
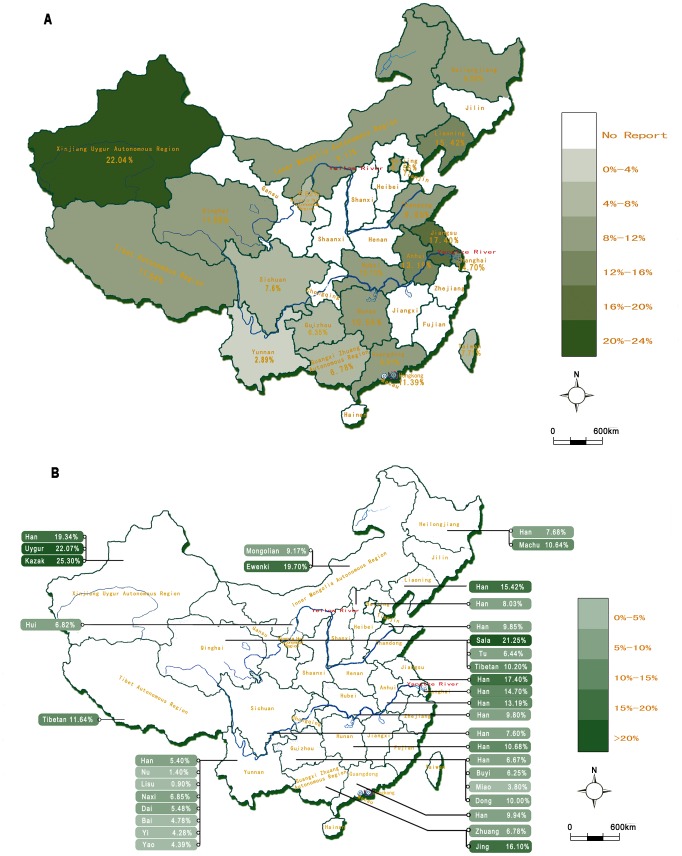
The frequency of DQB1*0201 or DQB1*0201/02 in Chinese populations. A: The DQB1*0201 or DQB1*0201/02 allele frequency in Chinese populations originated from inhabitants of 21 regions. The data from Jiangsu give the frequency of DQB1*0201/02, and the data from the other 20 regions are the frequency of DQB1*0201; B: The DQB1*0201 or DQB1*0201/02 allele frequencies of 21 ethnic minorities originated from nine geographic regions, and for the Han from 14 specific regions. The data for the Ewenki (Inner Mongolia), Dong (Guizhou), Miao (Guizhou), Jing (Guangxi), Naxi (Yunnan), Bai (Yunnan), Yao (Yunnan), and Han (Jiangsu) are the frequencies for DQB1*0201/02, and the others are the frequencies for DQB1*0201. The data sources are given in Table S3 and Table S4.

The DQB1*0201 allele frequency in Chinese populations in the different provinces, cities and autonomous regions is indicated on the map in Figure 5. Firstly, the results show that the DQB1*0201 allele is common in northwest China but relatively less common in the southwest populations. The allele frequency is especially high in those populations (22.04%) living in the Xinjiang Uygur Autonomous Region of northwestern China. In contrast, the frequency was the lowest (2.89%) in the populations living in Yunnan province, southwest China. The allele frequencies in the ethnic minorities showed a similar, decreasing trend from north to south China, except in the Hui, Tu and Jing minority groups. Secondly, the DQB1*02 allele frequency was similar in both Han subgroups and in the local ethnic minorities living in the same area. Thirdly, the 21 ethnic minorities originating from nine provinces or the Autonomous Region, the Kazak and Uygur living in northwest China have the highest occurrence of the DQB1*0201 allele. The weighted averages were 25.30% and 22.07%, respectively. In contrast, the DQB1*0201 allele appeared to be rare among the Lisu (0.9%) and Nu (1.4%) living in Yunnan in southwest China.

### Presence of gluten in the Chinese diet

**Figure 6:**
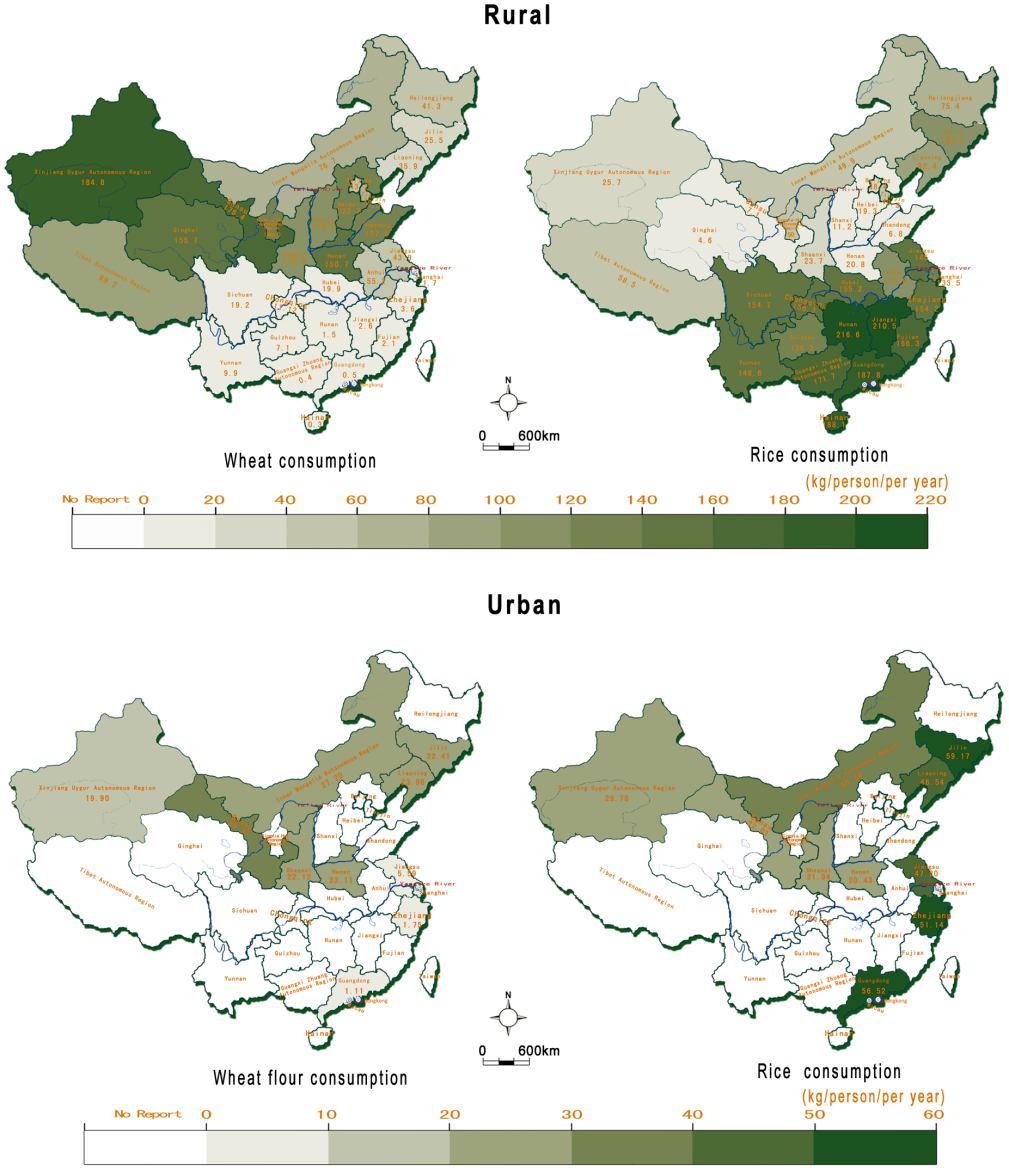
The per capita annual consumption of wheat and rice in rural households and urban households in 2009. In rural areas, the rice and wheat consumption are calculated on a dry, rough (unprocessed, unhusked or unmilled) weight basis. In urban households, the rice and wheat flour consumption are calculated on a processed (husked or milled) weight basis. Data sources (2010): China Yearbook of Rural Household Survey; Gansu Development Yearbook; Xinjiang Production & Construction Group Statistical Yearbook; Statistics yearbooks from different provinces (Inner Mongolia, Liaoning, Jilin, Henan, Shaanxi, Jiangsu, Zhejiang, Guangdong).

We obtained relevant data from 12 Chinese statistics yearbooks of 2010 on the consumption of wheat and rice in China. Wheat is the second staple food of the Chinese, after rice. In 2009, the per capita annual consumption of wheat in rural households and of wheat flour in urban households was 59.6 kg and 12.5 kg, respectively [32], [33]. The per capita annual consumption of wheat (or wheat flour) and of rice in rural households from 31 regions and urban households from ten provinces is shown in Figure 6. It shows that: (1) the consumption of wheat (or wheat flour) in the northern area is higher than in the southern area, where rice is the staple diet; (2) most of the rural households living in northern China consume more wheat than rice, except for the three northeastern provinces (Heilongjiang, Jilin, Liaoning), the provinces of Jiangsu and Anhui, and Shanghai city; and (3) rice is the staple diet for southern urban households. In the northern urban households, the consumption of wheat flour is slightly higher than or similar to the consumption of rice, except for the provinces of Jilin, Liaoning, and Jiangsu.

Regarding the consumption of gluten-containing food, relevant and reliable data were obtained from three Chinese statistics yearbooks. The consumption of gluten-containing convenience foods (such as bread, steamed bread, instant noodles) is increasing. The per capita annual purchases of breads and steamed breads in urban households were 1.14 kg and 6.82 kg, respectively in 2001, and it rose by 37.3% and 69.65%,respectively between 1995 and 2001[34], [35]. The per capita annual consumption of instant noodles was 34 packets (more than3 kg), and the total production of biscuits was over 2.8 million metric tons in 2008 [36].

## Discussion

This meta-analysis is, to our knowledge, the first elaborate study on the reported and potential occurrence of CD in China and may serve as a landmark reference point. We found several reported cases of CD in China and, based on HLA-DQ2.5 and-DQ8 genotyping, we suspect that CD is not rare in China, especially in the northern and northwestern parts where wheat consumption is common. Because the traditional rice-based diets are now being replaced by Western-style diets, with a significantly higher content of wheat and gluten, a rapid rise in the incidence of CD can be expected in China. This rise will occur especially in the southern urban regions, and among adolescents and young adults all over China. So far, we feel CD has been under-diagnosed by Chinese doctors, although there is a growing awareness about the occurrence of this disease and its relation to gluten consumption.

### Definite and suspected cases of CD

Currently, the gold standard of CD diagnosis is still a small bowel biopsy. The most characteristic histological markers in CD patients are atrophic villi, crypt hyperplasia, and intraepithelial lymphocyte infiltration. Since similar pathological changes are seen in several other diseases (e.g., tropical sprue, refractory sprue, Crohn's disease, radiation damage), and the extent and severity of the histological lesion is correlated to the intensity of the clinical symptoms, the histological damage is not pathognomonic of CD. Therefore, the number and the location of the biopsies procured interfere with correct histological diagnosis, and at least four duodenal biopsy samples must be obtained, three from the second part of the duodenum distal to the papilla, and one from the duodenal bulb. Moreover, small bowel biopsy does not always allow a correct diagnosis of CD. Nowadays, small bowel biopsy together with positive CD-specific serology is recommended as the gold standard for diagnosing CD. CD-specific serological tests include antibodies against gliadin (AGAs), anti-tissue transglutaminase (tTG) or anti-endomysial (EMA) tests. Regarding sensitivity and specificity, the anti-tTG and anti-EMA antibody tests are more often used for diagnostic purpose than the AGAs test, which is considered obsolete. Although the EMA test is considered the gold serological standard in CD, the test method is an indirect immunofluorescence approach, which is qualitative or semi-quantitative, expensive, time-consuming and operator-dependent. Enzyme-linked immunosorbent assay (ELISA) tests for IgA anti-tTG antibodies without the limitations of the EMA test are now widely available. However, there are differences between the different commercial anti-tTG antibody kits, and each laboratory should standardize the assay procedures, and should establish its own cut-off values. In addition, endoscopy, CD HLA genotyping, and gluten challenges are auxiliary diagnosis methods of CD [2].

In our study, 22 biopsy-confirmed CD cases have been reported [19]–[24], and suspected CD patients that had been diagnosed by serological testing for CD autoantibodies, GFD, or capsule endoscopy in patients displaying CD symptoms, were found in three publications [25]–[27] (Table 1). Among the eleven papers we have cited on definite and suspected CD cases [14], [19]–[28], eight were published in the last five years (2008–2012) [21]–[28]. This may reflect an increase in the incidence of CD, as well as a greater awareness among Chinese health professionals.

### Predisposing gene frequencies

Of the genes known to predispose to CD, the HLA-DQ2 antigen was more common in the northwestern than in the southeastern populations. The DQB1*0201 allele also appeared at a much higher frequency in northern China than in southern China, not only in the Han subgroups but also in the ethnic minorities. Additional subgroup analyses basically showed the same results, with only a minor inconsistency when comparing the DQB1*0201 allele between the Han subgroups and the ethnic minorities (Table S5). This indicated that the DQB1*0201 allele is significantly more common among the northern ethnic minorities (16.63%) than among the southern ethnic minorities (4.50%). When looking at the frequencies of the DQB1*0201 allele in the different ethnic minority subgroups, and when the different studies on northern and southern ethnic minorities were included and weighted in the statistics, the allele frequencies of the different ethnic minority subgroups appeared to be even more dissimilar, whereas the differences in the DQB1*0201 allele between the northern and southern Han were not significant.

Considering the frequency data from a regional point of view, the DQB1*0201 allele appeared to be highly prevalent in populations from Xinjiang Uygur Autonomous Region in northwest China (22.04%), whereas the allele was least common in populations living in Yunnan province in southwest China (2.89%). Studies [16], [18] on the frequency of various genetic loci and on anthropological characteristics clearly show the geographical and genetic boundary between Europeans and Asians running through northwestern China. Among the various Chinese populations, that in the northwest is more closely related to Europeans than the other populations in southern China. Caucasian genes flowed particularly into northern China, from west to east, and then from east to south. By way of comparison, the DQB1*02 allele is common in Europe (19.7%) (www.ncbi.nlm.nih.gov/projects/gv/mhc/ihwg.cgi), and its frequency in Xinjiang is in the same range. This migratory route would explain why the DQB1*02 allele is more common in the northern populations than southern populations. In addition, the allele frequency was similar in both the Han and the ethnic minorities living in the same area. This suggests a genetic flow between the Han and the ethnic minorities, which agrees with earlier data [17] that provided genetic proof that the Han subpopulations in different regions were genetically close to the local ethnic minorities. As a result, the Han population, which constitutes about 91% of the total Chinese population, should not be considered as an ethnic group with a high genetic kinship, but rather as a socio-cultural group. Exceptions were found in the Jing, Tu and Hui ethnic groups, where DQB1*02 was remarkably frequent in Jing living in Guangxi province in south China, but less common among Tu from Qinghai province and Hui from Ningxia in north China [37]–[39].

It is also important to mention the limitations of our meta-analysis of CD gene frequencies. Firstly, significant heterogeneity of the gene frequencies was found by studies in subgroups according to the nationality and place of residence of the participants. This was not unexpected in view of the different numbers of participants and the variance in precision associated with the different methods used for typing the HLA class ≥? alleles (such as PCR-SSO, PCR-RFLP, PCR-SSP and PCR-SBT). Even after further analysis of the studies with larger samples (100 subjects) and the four subgroups (PCR-SSO, PCR-RFLP, PCR-SSP and PCR-SBT) according to the different PCR-based DNA typing techniques separately, we still found a remarkable heterogeneity among the studies in most of the subgroups (Table S5). Secondly, the current place of residence is not always the same as the place of origin. Strictly speaking, only the gene frequencies of those participants whose grandparents lived at the same location can represent the frequencies of the population from that region. But only 20 out of the 112 studies clearly stated that all the participants and their families had lived in the same geographic area for at least three generations (Table S3 and Table S4). This could have affected the accuracy of the geographic distribution of the DQB1*02 allele frequency among the Chinese population groups. Thirdly, both HLA and non-HLA loci predispose to CD, but here we only analyzed the frequencies of HLA-DQ in China. In the future, we would need to check for the prevalence of the non-HLA loci as well, since this might indicate the transferability of the non-HLA genetic risk in China.

### Exposure to gluten

With the increased consumption of wheat, the overall exposure of the Chinese population to gluten has increased over the past 50 years, even in the southern populations where rice is still the staple diet. Wheat is the second staple food for the Chinese population after rice. China is the world's largest producer and consumer of wheat (especially in the northern area), and the national annual consumption of wheat is over 100 million metric tons for over 1,370 million population [40].

Exceptions in wheat consumption were found in the three northeastern provinces (Heilongjiang, Jilin, Liaoning), Jiangsu and Anhui provinces, and Shanghai city, where the population still consumes more rice than wheat. This might be related to the fact that rice is the main crop in these regions [41], and that the diet of the residents is directly connected with the local rice plantations.

The consumption of wheat and rice showed obvious differences between rural and urban households. The consumption of wheat flour and rice were similar in the major northern urban areas. There are more mobile populations in urban areas than in rural areas, and the diets of southern and northern populations will become more and more mixed, especially in the large cities like Shanghai [42]. Nowadays, in some southern cities, people are becoming increasingly used to eating wheat-based foods for breakfast (steamed bread, steamed stuffed bun, noodles). The rapidly changing diet, from rice- to wheat-based, may imply that genetically predisposed individuals are increasingly vulnerable to becoming overt CD patients.

Another factor provoking change in diet is the increasing demand for convenience foods (such as instant noodles, biscuits), which are made of wheat flour. This is accompanying the gradually improving quality and accelerating pace of urban life. At the same time, western nutrition patterns are spreading widely in China, providing more gluten-containing foods (e.g. bread, pasta). By June 2013, there were over 4,000 KFC restaurants and over 1,700 McDonalds in China [43].

Trying to estimate the exposure to gluten had several limitations. Firstly, the statistical data reported the consumption of wheat in rural households, whereas in urban households the data reported the consumption of wheat flour. This hindered making a balanced comparison between the exposure to gluten in both types of household. Secondly, there were few data on the consumption of wheat flour and rice in southern urban households. Thirdly, statistical data about the consumption of gluten-containing food are scarce. In the most of Chinese Statistical Yearbook databases, a statistical index is given for cake, a food product with wheat flour or rice flour as the main ingredient, which did not allow us to calculate the amount of gluten consumed. Fourthly, there were no relevant calculated data available to quantify the consumption or intake of gluten or gliadin in China directly. The consumption of gluten and gliadin can only be analyzed indirectly from the overall wheat consumption data.

### Strategies for prevention

Based on the data about HLA and wheat consumption, our study strongly suggests that the prevalence of CD is much higher in China than previously expected and likely to rise as the people adopt a more Western diet. This makes China a country at risk, for which strategies for primary and secondary prevention should be developed aiming at limiting the incidence of celiac disease and symptom severity [44]. Such strategies could include recommending an optimal infant diet, with emphasis on breastfeeding and weaning practices [4], [45]. Measures could also be taken to reduce or even eliminate the toxicity of wheat and its gluten for CD patients, while maintaining food quality [44], [46]–[48], for example, through developing advanced wheat varieties [49], [50] or using different food processing, like sourdough fermentation [51], [52]. In addition, for CD patients, gluten-free food production chains could be established to produce guaranteed safe foods, including alternative cereals such as oats [53], [54]. This requires specific legislation for detailed labelling of gluten-free food products in China, as has been made recently in Europe [55] and the U.S.A [56]. The Chinese medical and agricultural research organizations, food companies, and governmental health departments, together with international experts, could cooperate on all these prevention issues.

## Conclusions

Our analysis shows that the prevalence of CD might be more common in China than previously appreciated. Northern, and particularly the northwestern, Chinese populations may have a higher prevalence of CD than southern populations because of their genetic background. The northern groups have an HLA-related background, with apparent gene flows from Caucasian origin, and a diet that includes wheat as the staple food. Based on the rapidly changing dietary patterns in modern China, with increasing amounts of wheat and gluten being consumed, a sharp rise in the prevalence of CD can be expected nationwide. This may well justify mass population screening for genetic markers that predispose to CD, as well as coordinated measures from the government, medical and agricultural research institutions, and the food industries. Awareness of the health and societal impact of CD should be raised and they should be developing prevention strategies to guarantee the health and quality of life of Chinese individuals suffering from silent or active celiac disease.

## Supporting Information

Table S1 **Characteristics of included studies on HLA-DQ2.5 and HLA-DQ8 haplotypes in Chinese populations.** There were different resolution rates used by these studies. Jin 2011: DQA1*05-DQB1*0201; Trachtenberg 2007: DQA1*0300-DQB1*0302; Yu 2006: DQA1*0501-DQB1*0201/02; DQA1*0301/02/03-DQB1*0302; Wang 2007: DQA1*05-DQB1*0201; DQA1*03-DQB1*0302. Abbreviations: PCR-SSP, polymerase chain reaction-sequence specific primers; PCR-RFLP, polymerase chain reaction-restriction fragment length polymorphism; PCR-SSO, polymerase chain reaction-sequence specific oligonucleotide; PCR-SBT, polymerase chain reaction-sequence based typing. The data sources are given in Appendix S1.
(DOC)

Table S2 **Characteristics of included studies on HLA-DQ2 and HLA-DQ8 antigens in Chinese populations.** Abbreviations: PCR-SSP, polymerase chain reaction-sequence specific primers. The data sources are given in Appendix S1.
(DOC)

Table S3 Characteristics of included studies on HLA-DQB1*0201 allele frequency in Chinese populations. Abbreviations: PCR-SSP, polymerase chain reaction-sequence specific primers; PCR-RFLP, polymerase chain reaction-restriction fragment length polymorphism; PCR-SSO, polymerase chain reaction-sequence specific oligonucleotide; PCR-SBT, polymerase chain reaction-sequence based typing. The data sources are given in Appendix S1.
(DOC)

Table S4 Characteristics of included studies on HLA-DQB1*0201/02 allele frequency in Chinese populations. Abbreviations: PCR-SBT, polymerase chain reaction-sequence based typing; PCR-SSP, polymerase chain reaction-sequence specific primers; PCR-SSO, polymerase chain reaction-sequence specific oligonucleotide. The data sources are given in Appendix S1.
(DOC)

Table S5 Additional subgroup analyses of DQB1*0201 allele frequency. Abbreviations: CI, confidence interval; HG, heterogeneity; PCR-SSP, polymerase chain reaction-sequence specific primers; PCR-SSO, polymerase chain reaction-sequence specific oligonucleotide; PCR-SBT, polymerase chain reaction-sequence based typing; PCR-RFLP, polymerase chain reaction-restriction fragment length polymorphism.
(DOC)

Checklist S1 **PRISMA 2009 Checklist.**
(DOC)

Appendix S1 **References in Supplementary tables (Table S1-Table S4).**
(DOC)
