# Supplementary material for: The Tip of the “Celiac Iceberg” in China: A Systematic Review and Meta-Analysis
Source: PLoS One. 2013 Dec 4;8(12):e81151. doi: 10.1371/journal.pone.0081151 (PMC3852028; doi:10.1371/journal.pone.0081151)
Supplement: Table S1 — Characteristics of included studies on HLA-DQ2.5 and HLA-DQ8 haplotypes in Chinese populations. There were different resolution rates used by these studies. Jin 2011: DQA1*05-DQB1*0201; Trachtenberg 2007: DQA1*0300-DQB1*0302; Yu 2006: DQA1*0501-DQB1*0201/02; DQA1*0301/02/03-DQB1*0302; Wang 2007: DQA1*05-DQB1*0201; DQA1*03-DQB1*0302. Abbreviations: PCR-SSP, polymerase chain reaction-sequence specific primers; PCR-RFLP, polymerase chain reaction-restriction fragment length polymorphism; PCR-SSO, polymerase chain reaction-sequence specific oligonucleotide; PCR-SBT, polymerase chain reaction-sequence based typing. The data sources are given in Appendix S1. (DOC) [file pone.0081151.s001.doc]

**Table S1 Characteristics of included studies on HLA-DQ2.5 and HLA-DQ8 haplotypes in Chinese populations.**

|  |  |  | **Frequencies of HLA-DQ haplotypes n(%)** | |  |  |
| --- | --- | --- | --- | --- | --- | --- |
| **First author, year** | **Ethnic group/region** | **Number of subjects (male/female)** | **DQA1*0501-DQB1*0201** | **DQA1*0301-DQB1*0302** | **HLA typing method** | **Source of sample date** |
| Chen 1999 | /Taiwan | 65 | 8 (6.15) | 7 (5.38) | PCR-SSP | Controls for disease study |
| Ji 2005 | Han/Jiangsu | 45 (38/9) | 1 (1.11) | 1 (1.11) | PCR-SSP | Controls for disease study |
| Jin 2011 |  | 476 (297/179) | 31 (3.26) |  | PCR-SSP | Controls for disease study |
| Li 1998 | Han/North | 130 | 1 (0.38) | 2 (0.77) | PCR-SSP | Controls for disease study |
| Liu 1999 | Han | 50 (28/22) | 5 (5) |  | PCR-RFLP | Controls for disease study |
| Trachtenberg2007 | Han/South | 264 | 34 (6.4) | 37 (7.0) | PCR-SSO | Anthropology study |
| Tsai 2011 | /Taiwan | 268 (252/16) | 16 (2.99) |  | PCR-SSP | Controls for disease study |
| Wang 2007 | Han/Hunan | 226 | 16 (3.54) | 17 (3.76) | PCR-SSP | Controls for disease study |
| Xu 1992 | Buyi/Guizhou | 67 | 5 (7.46) | 5 (7.46) | PCR-SSO | Anthropology study |
| Yu 2006 | Han/Jiangsu | 160 (85/75) | 23 (7.19) | 15 (4.69) | PCR-SBT | Anthropology study |

There were different resolution rates used by these studies. Jin 2011: DQA1*05-DQB1*0201; Trachtenberg 2007: DQA1*0300-DQB1*0302; Yu 2006: DQA1*0501-DQB1*0201/02; DQA1*0301/02/03-DQB1*0302; Wang 2007: DQA1*05-DQB1*0201; DQA1*03-DQB1*0302.

Abbreviations: PCR-SSP, polymerase chain reaction-sequence specific primers; PCR-RFLP, polymerase chain reaction-restriction fragment length polymorphism; PCR-SSO, polymerase chain reaction-sequence specific oligonucleotide; PCR-SBT, polymerase chain reaction-sequence based typing. The data sources are given in Appendixe S1.
