# Supplementary material for: The Tip of the “Celiac Iceberg” in China: A Systematic Review and Meta-Analysis
Source: PLoS One. 2013 Dec 4;8(12):e81151. doi: 10.1371/journal.pone.0081151 (PMC3852028; doi:10.1371/journal.pone.0081151)
Supplement: Table S2 — Characteristics of included studies on HLA-DQ2 and HLA-DQ8 antigens in Chinese populations. Abbreviations: PCR-SSP, polymerase chain reaction-sequence specific primers. The data sources are given in Appendix S1. (DOC) [file pone.0081151.s002.doc]

**Table S2 Characteristics of included studies on HLA-DQ2 and HLA-DQ8 antigens in Chinese populations.**

|  |  |  | **Frequencies of HLA-DQ antigens** | |  |  |
| --- | --- | --- | --- | --- | --- | --- |
| **First author, year** | **Ethnic group/region** | **Number of subjects (male/female)** | **DQ2 n(%)** | **DQ8 n(%)** | **HLA typing method** | **Source of sample date** |
| Chen 1999 | Li/Hainan | 81 | 21 (25.93) |  | Microdrop lymphocyte cytotoxicity assay | Anthropology study |
| Er 1994 | Han/Beijing | 59 (19/40) |  | 11 (18.64) | Microdrop lymphocyte cytotoxicity assay | Anthropology study |
| Hwang 2002 | /Taiwan | 142 | 19 (13.38) |  | Microdrop lymphocyte cytotoxicity assay | Controls for disease study |
| Lin 1990 | /Taiwan | 107 (64/43) | 13 (12.15) |  | Microdrop lymphocyte cytotoxicity assay | Controls for disease study |
| Shaw 1997 | /Taiwan | 18206 | 3322 (18.25) |  | Microdrop lymphocyte cytotoxicity assay | Anthropology study |
| Si 1998 | Han/ | 49 (24/25) | 18 (36.73) |  | Microdrop lymphocyte cytotoxicity assay | Controls for disease study |
| Sun 1993 | /Taiwan | 130 (74/56) | 14 (10.77) |  | Microdrop lymphocyte cytotoxicity assay | Controls for disease study |
| Sun 2001 | /Taiwan | 310 (179/131) | 43 (13.87) |  | Microdrop lymphocyte cytotoxicity assay | Controls for disease study |
| Tang 1999 | Han/Shanghai | 185 | 49 (26.49) |  | Microdrop lymphocyte cytotoxicity assay | Controls for disease study |
| Xu 1993 | Buyi/Guizhou | 62 | 3 (4.84) |  | Microdrop lymphocyte cytotoxicity assay | Anthropology study |
| Zhang 1994 | Han/ | 100 (57/43) | 3 (3.00) |  | Microdrop lymphocyte cytotoxicity assay | Controls for disease study |
| Zhang 1997 | Han/South | 100 | 17 (17.00) |  | Microdrop lymphocyte cytotoxicity assay | Controls for disease study |
| Zhou 1994 | Han/Southeast | 285 | 51 (17.89) |  | Microdrop lymphocyte cytotoxicity assay | Controls for disease study |
| Chen 2004 |  | 100 (62/48) | 32 (32.00) | 15 (15.00) | PCR-SSP | Controls for disease study |
| Liang 2004 |  | 113 | 27 (23.89) | 6 (5.31) | PCR-SSP | Controls for disease study |
| Rudwaleit 1995 | /Singapore | 77 | 13 (16.88) | 8 (10.39) | PCR-SSP | Controls for disease study |
| Wang 2001 | - | 130 | 43 (33.07) | 5 (3.85) | PCR-SSP | Controls for disease study |
| Wang 2006 | /North | 58 | 14 (24.14) | 4 (6.90) | PCR-SSP | Controls for disease study |
| Zhai 2007 |  | 81 | 18 (22.22) | 4 (4.94) | PCR-SSP | Controls for disease study |

Abbreviations: PCR-SSP, polymerase chain reaction-sequence specific primers. The data sources are given in Appendixe S1.
