# Supplementary material for: The Tip of the “Celiac Iceberg” in China: A Systematic Review and Meta-Analysis
Source: PLoS One. 2013 Dec 4;8(12):e81151. doi: 10.1371/journal.pone.0081151 (PMC3852028; doi:10.1371/journal.pone.0081151)
Supplement: Table S3 — Characteristics of included studies on HLA-DQB1*0201 allele frequency in Chinese populations. Abbreviations: PCR-SSP, polymerase chain reaction-sequence specific primers; PCR-RFLP, polymerase chain reaction-restriction fragment length polymorphism; PCR-SSO, polymerase chain reaction-sequence specific oligonucleotide; PCR-SBT, polymerase chain reaction-sequence based typing. The data sources are given in Appendix S1. (DOC) [file pone.0081151.s003.doc]

**Table S3 Characteristics of included studies on HLA-DQB1*0201 allele frequency in Chinese populations.**

| **First author, year** | **Ethnic group/region** | **Number of subjects (male/female)** | **DQB1 * 0201 allele frequency n（%）** | **HLA typing method** | **Source of sample date** | **Family** |
| --- | --- | --- | --- | --- | --- | --- |
|  | **South** |  |  |  |  |  |
| Du 2006 | Han/Guangdong | 105 | 23 (10.95) | PCR-SSP | Controls for disease study |  |
| Gong 2010 | Han/Guangdong | 454 (230/224) | 94（10.35) | PCR-SSP | Controls for disease study |  |
| Sun 1997 | Han/Guangdong | 102 | 16（7.84) | PCR-SSO | Anthropology study |  |
| Zha 2004 | Han/Guangdong | 110 | 24 (10.91) | PCR-SSP | Controls for disease study | Grandparents live at same location |
| Zhu 2009 | Han/Guangdong | 144 (82/62) | 25 (8.68) | PCR-SBT | Anthropology study |  |
| Liang 2008 | Han/Guizhou | 30 | 4 (6.67) | PCR-SSP | Controls for disease study |  |
| Gong 1999 | Han/Hubei | 160 | 32 (10) | PCR-RFLP | Anthropology study |  |
| Lin 2001 | Han/Hubei | 136 (62/74) | 26 (9.56) | PCR-SSP | Controls for disease study |  |
| Lin 2002 | Han/Hubei | 128 | 26 (10.16) | PCR-SSP | Controls for disease study |  |
| Zhang 2000 | Han/Hubei | 183 | 35 (9.56) | PCR-SSP | Controls for disease study |  |
| Wang 2007 | Han/Hunan | 248 | 53 (10.68) | PCR-SSP | Controls for disease study |  |
| Jiang 2003 | Han/Sichuan | 106 (88/18) | 23 (10.85) | PCR-SSP | Controls for disease study |  |
| Liu 2004 | Han/Sichuan | 52 (29/23) | 1 (0.96) | PCR-SSP | Controls for disease study |  |
| Wen 2005 | Han/Yunnan | 37 | 4 (5.40) | PCR-SSP | Controls for disease study |  |
| Wu 2011 | Han/Jiangxi and Fujian | 48 (15/33) | 6 (6.25) | PCR-SBT | Controls for disease study |  |
| Gao 2009 | Han/South | 186 | 12 (3.22) | PCR-SBT | Anthropology study |  |
| Li 1996 | Han/South | 75 | 24 (16) | PCR-SSO | Controls for disease study |  |
| Sun 1992 | Han/South | 135 | 27 (10) | PCR-SSO | Anthropology study |  |
| Trachtenberg 2007 | Han/South | 264 | 64 (12.12) | PCR-SSO | Anthropology study |  |
| Chen 2007 | Lisu/Yunnan | 111 | 2 (0.90) | PCR-SSP | Anthropology study |  |
| Chen 2007 | Nu/Yunnan | 107 | 3 (1.4) | PCR-SSP | Anthropology study |  |
| Fan 1992 | Dai/Yunnan | 73 | 8 (5.48) | PCR-SSO | Anthropology study |  |
| Wen 2004 | Yi/Yunnan | 70 | 6 (4.28) | PCR-SSP | Anthropology study |  |
| Liu 2011 | Buyi/Guizhou | 96 (60/36) | 12 (6.25) | PCR-SSP | Anthropology study | Grandparents live at same location |
| Long 1998 | Zhuang/Guangxi | 140 | 10 (3.57) | PCR-SSP | Controls for disease study | Grandparents live at same location |
| Long 2000 | Zhuang/Guangxi | 71 (30/41) | 13 (9.15) | PCR-SSP | Anthropology study | Grandparents live at same location |
| Pan 2005 | Zhuang/Guangxi | 143 (64/79) | 25 (8.74) | PCR-SSP | Anthropology study | Grandparents live at same location |
| Chen 2002 | /Guangdong | 63 | 11 (8.73) | PCR-SSO | Controls for disease study |  |
| Chowdari 2001 | /Guangdong | 86 | 18 (10.46) | PCR-SSP | Controls for disease study |  |
| Wang 2000 | /Hubei | 143 (98/45) | 33 (11.54) | PCR-SSP | Controls for disease study |  |
| Lu 1997 | /Taiwan | 115 | 18 (7.83) | PCR-SSO | Controls for disease study |  |
| Chen 1999 | /Taiwan | 65 | 10 (7.69) | PCR-SSP | Controls for disease study |  |
| Huang 1995 | /Taiwan | 205 | 25 (6.10) | PCR-SSP | Controls for disease study |  |
| Tsai 2011 | /Taiwan | 268 | 54 (10.07) | PCR-SSP | Controls for disease study |  |
| Yang 2009 | /Taiwan | 183 | 22 (6.01) | PCR-SSP | Anthropology study |  |
| Yu 1999 | /South | 75 | 14 (9.33) | PCR-SSO | Controls for disease study |  |
| Chang 1998 | /Hongkong | 250 | 63（12.6） | PCR-SSP | Controls for disease study |  |
| Donaldson 2001 | /Hongkong | 123 | 22 (8.94) | PCR-SSO | Controls for disease study |  |
| Chan 1994 | /South | 80 | 14 (8.75) | PCR-SSO | Controls for disease study |  |
| Li 1995 | /South | 78 | 14（8.97） | PCR-SSP | Controls for disease study |  |
|  | **North** |  |  |  |  |  |
| Xiao 2005 | Han/Anhui | 273 (140/133) | 72 (13.19) | PCR-SSP | Controls for disease study |  |
| Gao 2002 | Han/Beijing | 96 (63/33) | 19 (9.90) | PCR-SSP | Controls for disease study | Grandparents live at same location |
| Wang 2004 | Han/Beijing | 41 (22/19) | 3 (3.66) | PCR-SSP | Controls for disease study |  |
| Huang 2004 | Han/Heilongjiang | 115 (60/55) | 29 (12.61) | PCR-SSP | Controls for disease study |  |
| Liu 2005 | Han/Heilongjiang | 100 (62/38) | 4 (2.00) | PCR-SSP | Controls for disease study |  |
| Zhou 2002 | Han/Jiangsu and Anhui | 53 | 18 (16.98) | PCR-SSP | Controls for disease study |  |
| Yu 1995 | Han/Liaoning | 94 | 29 (15.42) | PCR-SSO | Anthropology study | Grandparents live at same location |
| Zhou 2007 | Han/Shandong | 70 (37/33) | 20 (14.28) | PCR-SSP | Controls for disease study |  |
| Zhou 2005 | Han/Shandong | 60 | 19 (15.83) | PCR-SSP | Controls for disease study |  |
| Xing 2001 | Han/Shandong | 50 (24/26) | 8 (8) | PCR-SBT | Controls for disease study |  |
| Fu 2005 | Han/Shandong | 94 (58/36) | 7 (3.72) | PCR-SSP | Controls for disease study |  |
| Zhang 1996 | Han/Shanghai | 70 | 27 (19.28) | PCR-SSO | Controls for disease study |  |
| Kelly 1995 | Han/Shanghai | 87 | 21 (12.07) | PCR-RFLP | Controls for disease study |  |
| Guo 2001 | Han/Shanghai | 51 | 16 (15.68) | PCR-SSP | Controls for disease study |  |
| Xie 2000 | Han/Shanghai | 48 | 13 (13.54) | PCR-SSP | Controls for disease study |  |
| Jin 2003 | Han/Shanghai | 85 | 22 (12.94) | PCR-SSO | Controls for disease study |  |
| Xu 1999 | Han/Shanghai | 96 | 22 (11.46) | PCR-SSO | Controls for disease study |  |
| Zhong 2005 | Han/Shanghai | 80 | 31 (19.38) | PCR-SSO | Controls for disease study |  |
| Mizuki 1997 | Han/Xinjiang | 59 | 22 (18.64) | PCR-RFLP | Anthropology study |  |
| Zhang 1996 | Han/Xinjiang | 47 | 19 (20.21) | PCR-RFLP | Anthropology study | Grandparents live at same location |
| Geng 2000 | Han/Northeast | 160 | 32 (10) | PCR-SSP | Controls for disease study |  |
| Guo 2003 | Han/North | 91 | 27 (14.84) | PCR-SSO | Controls for disease study |  |
| Sang 1997 | Han/North | 40 | 10 (12.50) | PCR-SSP | Controls for disease study |  |
| Sun 1992 | Han/North | 171 | 43 (12.57) | PCR-SSO | Anthropology study |  |
| Wang 2007 | Han/North | 102 | 21 (10.29) | PCR-SSP | Controls for disease study |  |
| Geng 2005 | Sala/Qinghai | 80 (43/37) | 34 (21.25) | PCR-SSO | Anthropology study | Grandparents live at same location |
| Geng 2005 | Tu/Qinghai | 132 (71/61) | 17 (6.44) | PCR-SSO | Anthropology study | Grandparents live at same location |
| Li 1998 | Tibetan/Qinghai | 49 (25/24) | 10 (10.20) | PCR-RFLP | Anthropology study | Grandparents live at same location |
| Mizuki 1997 | Kazaks/Xinjiang | 42 | 20 (23.81) | PCR-RFLP | Anthropology study |  |
| Shen 1997 | Uygur/Xinjiang | 92 | 46 (25) | PCR-SSO | Anthropology study | Grandparents live at same location |
| Sun 1992 | Uygur/Xinjiang | 92 | 50 (27.17) | PCR-SSO | Anthropology study |  |
| Wang 2010 | Uygur/Xinjiang | 231 (118/113) | 80 (17.32) | PCR-SSP | Controls for disease study |  |
| Zhang 1998 | Kazaks/Xinjiang | 41 | 22 (26.83) | PCR-SSP | Anthropology study | Grandparents live at same location |
| Zhang 1998 | Uygur/Xinjiang | 54 | 31 (28.70) | PCR-SSP | Anthropology study | Grandparents live at same location |
| Geng 1995 | Manchu/Heilongjiang | 47 | 10 (10.64) | PCR-SSO | Anthropology study |  |
| Li 2011 | Tibetan/Tibet | 189 (112/77) | 44 (11.64) | PCR-SSP | Controls for disease study |  |
| Li 2012 | Mongolian/Inner Mongolia | 60 (34/26) | 11 (9.17) | PCR-SSP | Controls for disease study | Grandparents live at same location |
| Xu 2009 | Hui/Ningxia | 66 (30/36) | 9 (6.82) | PCR-SSP | Controls for disease study | Grandparents live at same location |
| Magira 2003 | /North | 97 | 6 (3.09) | PCR-SSP | Controls for disease study |  |
| Lu 2009 | /Beijing | 107 (57/50) | 10 (4.67) | PCR-SSP | Controls for disease study |  |
| Yu 2008 | /Jiangsu and Anhui | 160 | 57 (17.81) | PCR-SBT | Controls for disease study |  |
| Zhang 2001 | /Heilongjiang | 75 (40/35) | 21 (14.00) | PCR-SSO | Controls for disease study |  |

Abbreviations: PCR-SSP, polymerase chain reaction-sequence specific primers; PCR-RFLP, polymerase chain reaction-restriction fragment length polymorphism; PCR-SSO, polymerase chain reaction-sequence specific oligonucleotide; PCR-SBT, polymerase chain reaction-sequence based typing. The data sources are given in Appendixe S1.
