# Supplementary material for: The Tip of the “Celiac Iceberg” in China: A Systematic Review and Meta-Analysis
Source: PLoS One. 2013 Dec 4;8(12):e81151. doi: 10.1371/journal.pone.0081151 (PMC3852028; doi:10.1371/journal.pone.0081151)
Supplement: Table S4 — Characteristics of included studies on HLA-DQB1*0201/02 allele frequency in Chinese populations. Abbreviations: PCR-SBT, polymerase chain reaction-sequence based typing; PCR-SSP, polymerase chain reaction-sequence specific primers; PCR-SSO, polymerase chain reaction-sequence specific oligonucleotide. The data sources are given in Appendix S1. (DOC) [file pone.0081151.s004.doc]

**Table S4 Characteristics of included studies on HLA-DQB1*0201/02 allele frequency in Chinese populations.**

| **First author, Year** | **Ethnic group /Region** | **Number of subjects** | **DQB1*0201/02 allele frequency**  **n（%）** | **HLA typing method** | **Source of sample date** | **Family** |
| --- | --- | --- | --- | --- | --- | --- |
| Fu 2003 | Naxi/Yunnan | 73 | 10 (6.85) | PCR-SBT | Anthropology study |  |
| Hu 2008 | Bai/Yunnan | 128 | 12 (4.69) | PCR-SBT | Anthropology study |  |
| Huang 2007 | Bai/Yunnan | 70 | 6 (4.28) | PCR-SSP | Anthropology study |  |
| Wang 2009 | Bai/Yunnan | 124 | 14 (5.64) | PCR-SSP | Anthropology study |  |
| Yang 2007 | Bai/Yunnan | 34 | 2 (2.94） | PCR-SSP | Controls for disease study |  |
| Li 2008 | Dong/Guizhou | 40 | 8 (10) | PCR-SBT | Anthropology study | Grandparents live at same location |
| Liu 2006 | Miao/Guizhou | 79 | 6 (3.80) | PCR-SBT\PCR-SSP | Anthropology study | Grandparents live at same location |
| Lin 2003 | Jing/Guangxi | 118 | 38 (16.10) | PCR-SBT | Anthropology study |  |
| Liu 2006 | Yao/Yunnan | 57 | 5 (4.39) | PCR-SBT\PCR-SSP | Anthropology study | Grandparents live at same location |
| Su 2007 | Ewenki/Inner Mongolia | 94 | 37 (19.7) | PCR-SBT | Anthropology study |  |
| Waine 1998 | Han/Jiangsu | 44 | 14 (15.91) | PCR-SSO | Controls for disease study |  |
| Yu 2006 | Han/Jiangsu | 160 | 57 (17.81) | PCR-SBT | Anthropology study | Grandparents live at same location |

Abbreviations: PCR-SBT, polymerase chain reaction-sequence based typing; PCR-SSP, polymerase chain reaction-sequence specific primers; PCR-SSO, polymerase chain reaction-sequence specific oligonucleotide. The data sources are given in Appendixe S1.
