# Supplementary material for: The Tip of the “Celiac Iceberg” in China: A Systematic Review and Meta-Analysis
Source: PLoS One. 2013 Dec 4;8(12):e81151. doi: 10.1371/journal.pone.0081151 (PMC3852028; doi:10.1371/journal.pone.0081151)
Supplement: Table S5 — Additional subgroup analyses of DQB1*0201 allele frequency. Abbreviations: CI, confidence interval; HG, heterogeneity; PCR-SSP, polymerase chain reaction-sequence specific primers; PCR-SSO, polymerase chain reaction-sequence specific oligonucleotide; PCR-SBT, polymerase chain reaction-sequence based typing; PCR-RFLP, polymerase chain reaction-restriction fragment length polymorphism. (DOC) [file pone.0081151.s005.doc]

**Table S5 Additional subgroup analyses of DQB1*0201 allele frequency**

| **Subgroups** | **Studies (n)** | **Subjects (n)** | **Frequency (95%CI)** | **HG I^2^ (%)** | **HG p value** |
| --- | --- | --- | --- | --- | --- |
| **Subjects and their families have lived in the same geographic area for at least three generations** |  |  |  |  |  |
| Chinese | 16 | 1371 | 12.69% (9.52-15.84%) | 85.9 | <0.001 |
| Southern populations | 5 | 560 | 7.39% (4.52-10.26%) | 68.0 | 0.014 |
| Northern populations | 11 | 811 | 15.62% (11.09--20.16%) | 85.4 | <0.001 |
| Han subpopulations | 4 | 347 | 13.07% (9.26-16.88%) | 50.3 | 0.11 |
| Southern Han subpopulations | 1 | 110 | 10.91% (6.56-15.25%) | - | - |
| Northern Han subpopulations | 3 | 237 | 14.34% (8.79-19.89%) | 62.3 | 0.07 |
| Ethnic minorities | 12 | 1024 | 12.47% (8.60-16.34%) | 88.1 | <0.001 |
| Southern ethnic minorities | 4 | 450 | 6.55% (3.71-9.39%) | 62.0 | 0.048 |
| Northern ethnic minorities | 8 | 574 | 16.15% (9.99-22.30%) | 88.8 | <0.001 |
| **Group size ≥100** |  |  |  |  |  |
| Chinese | 36 | 5989 | 9.08% (7.60-10.56%) | 88.2 | <0.001 |
| Southern populations | 25 | 4249 | 8.35% (6.71-9.99%) | 87.6 | <0.001 |
| Northern populations | 11 | 1740 | 10.76% (7.97-13.55%) | 84.7 | <0.001 |
| Han subpopulations | 20 | 3382 | 9.74% (8.31-11.17%) | 72.3 | <0.001 |
| Southern Han subpopulations | 14 | 2461 | 9.51% (7.90-11.12%) | 71.2 | <0.001 |
| Northern Han subpopulations | 6 | 921 | 10.25% (7.16-13.33%) | 74.5 | 0.001 |
| Ethnic minorities | 7 | 1053 | 6.97% (2.99-10.95%) | 94.3 | <0.001 |
| Southern ethnic minorities | 4 | 501 | 3.30% (0.61-6.00%) | 83.8 | <0.001 |
| Northern ethnic minorities | 3 | 552 | 11.76% (5.61-17.91%) | 90.1 | <0.001 |
| **Different the PCR-based DNA typing techniques** |  |  |  |  |  |
| **PCR-SSP** |  |  |  |  |  |
| Chinese | 48 | 5753 | 9.10% (7.69-10.52%) | 86.3 | <0.001 |
| Southern populations | 26 | 3605 | 7.72% (6.05-9.39%) | 86.3 | <0.001 |
| Northern populations | 22 | 2148 | 11.12% (8.68-13.56%) | 84.3 | <0.001 |
| Han subpopulations | 25 | 2892 | 9.61% (7.94-11.29%) | 75.4 | <0.001 |
| Southern Han subpopulations | 11 | 1589 | 8.79% (6.52-11.06%) | 77.1 | <0.001 |
| Northern Han subpopulations | 14 | 1303 | 10.48% (7.89-13.08%) | 75.4 | <0.001 |
| Ethnic minorities | 13 | 1379 | 9.25% (5.90-12.60%) | 92.5 | <0.001 |
| Southern ethnic minorities | 7 | 738 | 4.41% (2.12-6.69%) | 79.7 | <0.001 |
| Northern ethnic minorities | 6 | 641 | 15.60% (10.14-21.05%) | 85.2 | <0.001 |
| **PCR-SSO** |  |  |  |  |  |
| Chinese | 23 | 2310 | 12.74% (10.70-14.79%) | 76.8 | <0.001 |
| Southern populations | 10 | 1105 | 9.40% (8.12-10.69%) | 30.9 | 0.162 |
| Northern populations | 13 | 1205 | 15.83% (12.51-19.16%) | 80.3 | <0.001 |
| Han subpopulations | 11 | 1263 | 12.57% (11.22-13.91%) | 46.3 | 0.045 |
| Southern Han subpopulations | 4 | 576 | 10.97% (9.09-12.86%) | 48.8 | 0.119 |
| Northern Han subpopulations | 7 | 687 | 14.24% (12.31-16.17%) | 15.9 | 0.309 |
| Ethnic minorities | 6 | 516 | 15.70% (8.09-23.31%) | 92.3 | <0.001 |
| Southern ethnic minorities | 1 | 73 | 5.48% (1.43-9.54%) | - | - |
| Northern ethnic minorities | 5 | 443 | 17.91% (8.72-27.10%) | 92.5 | <0.001 |
| **PCR-SBT** |  |  |  |  |  |
| Chinese | 5 | 588 | 8.71% (3.45-13.97%) | 89.7 | <0.001 |
| Southern populations | 3 | 378 | 5.82% (1.97-9.67%) | 74.1 | 0.021 |
| Northern populations | 2 | 210 | 13.11% (3.50-22.71%) | 85.8 | 0.008 |
| **PCR-RFLP** |  |  |  |  |  |
| Chinese | 6 | 444 | 14.65% (10.45-18.85%) | 63.2 | 0.018 |
| Southern populations | 1 | 160 | 10% (6.56-13.44%) | - | - |
| Northern populations | 5 | 284 | 16.10% (11.29-20.91%) | 55.4 | 0.062 |

Abbreviations: CI, confidence interval; HG, heterogeneity; PCR-SSP, polymerase chain reaction-sequence specific primers; PCR-SSO, polymerase chain reaction-sequence specific oligonucleotide; PCR-SBT, polymerase chain reaction-sequence based typing; PCR-RFLP, polymerase chain reaction-restriction fragment length polymorphism.
