# Supplementary material for: The Tip of the “Celiac Iceberg” in China: A Systematic Review and Meta-Analysis
Source: PLoS One. 2013 Dec 4;8(12):e81151. doi: 10.1371/journal.pone.0081151 (PMC3852028; doi:10.1371/journal.pone.0081151)
Supplement: Appendix S1 — References in Supplementary tables (Table S1-Table S4). (DOC) [file pone.0081151.s007.doc]

**Appendixe S1 References in Supplementary tables (Table S1-Table S4)**

[Chan SH](http://www.ncbi.nlm.nih.gov/pubmed?term=Chan SH%5BAuthor%5D&cauthor=true&cauthor_uid=8055196), [Lin YN](http://www.ncbi.nlm.nih.gov/pubmed?term=Lin YN%5BAuthor%5D&cauthor=true&cauthor_uid=8055196), [Wee GB](http://www.ncbi.nlm.nih.gov/pubmed?term=Wee GB%5BAuthor%5D&cauthor=true&cauthor_uid=8055196), [Koh WH](http://www.ncbi.nlm.nih.gov/pubmed?term=Koh WH%5BAuthor%5D&cauthor=true&cauthor_uid=8055196), [Boey ML](http://www.ncbi.nlm.nih.gov/pubmed?term=Boey ML%5BAuthor%5D&cauthor=true&cauthor_uid=8055196).(1994) HLA class II genes in Singaporean Chinese rheumatoid arthritis. Br J Rheumatol 33:713–717.

Chang YW, Lam KS, Hawkins BR. (1998) Strong association between DQA1/DQB1 genotype and early-onset IDDM in Chinese: the association is with alleles rather than specific residues. Eur J Immunogenet 25:273–280.

[Chen BH](http://www.ncbi.nlm.nih.gov/pubmed?term=Chen BH%5BAuthor%5D&cauthor=true&cauthor_uid=10600012), [Chiang CH](http://www.ncbi.nlm.nih.gov/pubmed?term=Chiang CH%5BAuthor%5D&cauthor=true&cauthor_uid=10600012), [Lin SR](http://www.ncbi.nlm.nih.gov/pubmed?term=Lin SR%5BAuthor%5D&cauthor=true&cauthor_uid=10600012), [Chao MG](http://www.ncbi.nlm.nih.gov/pubmed?term=Chao MG%5BAuthor%5D&cauthor=true&cauthor_uid=10600012), [Tsai ST](http://www.ncbi.nlm.nih.gov/pubmed?term=Tsai ST%5BAuthor%5D&cauthor=true&cauthor_uid=10600012).(1999) The influence of age at onset and gender on the HLA-DQA1, DQB1 association in Chinese children with insulin-dependent diabetes mellitus. Hum Immunol 60:1131–1137.

Chen DM, Li S, Bao WH. (2004) Study on correlation between HLA class II gene and keloids (in Chinese). Jie Pou Xue Bao 27:589-591.

[Chen S](http://www.ncbi.nlm.nih.gov/pubmed?term=Chen S%5BAuthor%5D&cauthor=true&cauthor_uid=17509456), [Hu Q](http://www.ncbi.nlm.nih.gov/pubmed?term=Hu Q%5BAuthor%5D&cauthor=true&cauthor_uid=17509456), [Xie Y](http://www.ncbi.nlm.nih.gov/pubmed?term=Xie Y%5BAuthor%5D&cauthor=true&cauthor_uid=17509456), [Zhou L](http://www.ncbi.nlm.nih.gov/pubmed?term=Zhou L%5BAuthor%5D&cauthor=true&cauthor_uid=17509456), [Xiao C](http://www.ncbi.nlm.nih.gov/pubmed?term=Xiao C%5BAuthor%5D&cauthor=true&cauthor_uid=17509456), et al. (2007) Origin of Tibeto-Burman speakers: Evidence from HLA allele distribution in Lisu and Nu inhabiting Yunnan of China. Hum Immunol 68:550–559.

Chen SQ, Deng WY, Qiang YG. (1999) Survey of HLA antigens in Li ethnic group in Hainan Province (in Chinese). Zhongguo Shu Xue Za Zhi 12:34-35.

Chen WM, Huang HL, Pan DJ, Liu ZH, Li JH, et al. (2002) Association between HLA-DQB1 gene with hepatitis B virus infection in southern Chinese (in Chinese). Zhongguo Bing Li Sheng Li Za Zhi 18:1041,1045.

[Chowdari KV](http://www.ncbi.nlm.nih.gov/pubmed?term=Chowdari KV%5BAuthor%5D&cauthor=true&cauthor_uid=11423178), [Xu K](http://www.ncbi.nlm.nih.gov/pubmed?term=Xu K%5BAuthor%5D&cauthor=true&cauthor_uid=11423178), [Zhang F](http://www.ncbi.nlm.nih.gov/pubmed?term=Zhang F%5BAuthor%5D&cauthor=true&cauthor_uid=11423178), [Ma C](http://www.ncbi.nlm.nih.gov/pubmed?term=Ma C%5BAuthor%5D&cauthor=true&cauthor_uid=11423178), [Li T](http://www.ncbi.nlm.nih.gov/pubmed?term=Li T%5BAuthor%5D&cauthor=true&cauthor_uid=11423178), et al. (2001) Immune related genetic polymorphisms and schizophrenia among the Chinese. Hum Immunol 62:714–724.

[Donaldson PT](http://www.ncbi.nlm.nih.gov/pubmed?term=Donaldson PT%5BAuthor%5D&cauthor=true&cauthor_uid=11318984), [Ho S](http://www.ncbi.nlm.nih.gov/pubmed?term=Ho S%5BAuthor%5D&cauthor=true&cauthor_uid=11318984), [Williams R](http://www.ncbi.nlm.nih.gov/pubmed?term=Williams R%5BAuthor%5D&cauthor=true&cauthor_uid=11318984), [Johnson PJ](http://www.ncbi.nlm.nih.gov/pubmed?term=Johnson PJ%5BAuthor%5D&cauthor=true&cauthor_uid=11318984). (2001) HLA class II alleles in Chinese patients with hepatocellular carcinoma. Liver 21:143–148.

Du YP, Ye HJ, Long X, Yu J, Fang JZ, et al. (2006) A study on HLA-DQBl allele associated with genetic susceptibility to duodenal ulcerin Guangdong Hans (in Chinese). Zhonghua Yi Xue Yi Chuan Xue Za Zhi 23:440-442.

Er XJ, Li ZX, Jia BX, Bi RM, Xu XH, et al. (1994) Polymorphism of HLA class Ⅰ, Ⅱ antigen in Beijing Han population (in Chinese). Zhongguo Shu Xue Za Zhi 7:96-97.

Fan LA, Yang YQ, Dong JZ, Yao FJ, Xu LD, et al. (1992) [HLA class II oligonucleotide typing of a Dai minority population in Xishuangbanna of Chian] (in Chinese). Zhongguo Mian Yi Xue Za Zhi 8:287–290.

[Fu Y](http://www.ncbi.nlm.nih.gov/pubmed?term=Fu Y%5BAuthor%5D&cauthor=true&cauthor_uid=12694588), [Liu Z](http://www.ncbi.nlm.nih.gov/pubmed?term=Liu Z%5BAuthor%5D&cauthor=true&cauthor_uid=12694588), [Lin J](http://www.ncbi.nlm.nih.gov/pubmed?term=Lin J%5BAuthor%5D&cauthor=true&cauthor_uid=12694588), [Jia Z](http://www.ncbi.nlm.nih.gov/pubmed?term=Jia Z%5BAuthor%5D&cauthor=true&cauthor_uid=12694588), [Chen W](http://www.ncbi.nlm.nih.gov/pubmed?term=Chen W%5BAuthor%5D&cauthor=true&cauthor_uid=12694588), et al. (2003) HLA-DRB1, DQB1 and DPB1 polymorphism in the Naxi ethnic group of south-western China. Tissue Antigens 61:179–183.

Fu HJ, Zhang Fr. (2005) The Association of H LA-DQB1 Alleles and Generailzed Pustular Psoriasis (in Chinese). Zhonghua Pi Fu Ke Za Zhi 38:137–139.

Gao JM, Lin YG, Qiu CC, Liu YW, Ma Y, et al. (2002) Relationship between HLA-DQA1,-DQB1 genes polymorphism and susceptibilty to bronchial asthma among Northern Hans (in Chinese) Zhonghua Yi Xue Za Zhi 82:379–383.

Gao SQ, Zou HY, Cheng LH, Jiang SZ, Deng ZH. (2009) Analysis on haplotypes of five HLA loci in southern Chinese Han population by sequence-based typing (in Chinese). Zhonghua Yi Xue Yi Chuan Xue Za Zhi 26:228–232.

[Geng L](http://www.ncbi.nlm.nih.gov/pubmed?term=Geng L%5BAuthor%5D&cauthor=true&cauthor_uid=7482503), [Imanishi T](http://www.ncbi.nlm.nih.gov/pubmed?term=Imanishi T%5BAuthor%5D&cauthor=true&cauthor_uid=7482503), [Tokunaga K](http://www.ncbi.nlm.nih.gov/pubmed?term=Tokunaga K%5BAuthor%5D&cauthor=true&cauthor_uid=7482503), [Zhu D](http://www.ncbi.nlm.nih.gov/pubmed?term=Zhu D%5BAuthor%5D&cauthor=true&cauthor_uid=7482503), [Mizuki N](http://www.ncbi.nlm.nih.gov/pubmed?term=Mizuki N%5BAuthor%5D&cauthor=true&cauthor_uid=7482503), et al. (1995) Determination of HLA class II alleles by genotyping in a Manchu population in the northern part of China and its relationship with Han and Japanese populations. [Tissue Antigens](javascript:AL_get(this, 'jour', 'Tissue Antigens.');) 46:111–116.

Geng L, Zhai N, Xiao T, Wang LM, Song FJ, et al. (2000) Association between HLA-DQB1 alleles and pemphigus vulgaris in the Han of north China (in Chinese). Zhonghua Pi Fu Ke Za Zhi 33:277.

Geng PL, Wu HF, Zhu HH, He JX, Zhao JD, et al. (2005) Investigaion of HLA-DQA1 and DQB1 polymorphism in TU and Sala nationalities of province (in Chinese). Zhongguo Mian Yi Xue Za Zhi 21:35–39.

Gong FL, Xiong P, Yang ZZ, Xu Y, Jiang XD,et al. (1999) An investigation of the polymorphism of HLA class II alleles in the Han population in Hubei province of China (in Chinese). Zhonghua Yi Xue Yi Chuan Xue Za Zhi 16:216–219.

Gong YN, Liu YL, Hu CH, Xiong YB, Huang WW, et al. (2010) Study on the relationship between HLA -DQB1 and susceptibility of type1 diabetes mellitus in Hans nationality Chinese of Guangdong province (in Chinese). Shi Yong Yi Xue Za Zhi 26:1577–1579.

Guo XJ, Ni PH, Li L ,Yu SC, Li YZ, et al. (2001) Association between asthma and the polymorphism of HLA-DQ genes (in Chinese). Zhonghua Jie He He Hu Xi Za Zhi 24:139-141.

Guo YH, Zou LP, Fang F, Jiang ZF. (2003) The potential association between HLA and idiopathic childhood stroke in Han Chinese (in Chinese). Zhonghua Wei Sheng Wu Xue He Mian Yi Xue Za Zhi 23:219-221.

[Hu W](http://www.ncbi.nlm.nih.gov/pubmed?term=Hu W%5BAuthor%5D&cauthor=true&cauthor_uid=18778327), [Tang L](http://www.ncbi.nlm.nih.gov/pubmed?term=Tang L%5BAuthor%5D&cauthor=true&cauthor_uid=18778327), [Wang J](http://www.ncbi.nlm.nih.gov/pubmed?term=Wang J%5BAuthor%5D&cauthor=true&cauthor_uid=18778327), [Wang B](http://www.ncbi.nlm.nih.gov/pubmed?term=Wang B%5BAuthor%5D&cauthor=true&cauthor_uid=18778327), [Li S](http://www.ncbi.nlm.nih.gov/pubmed?term=Li S%5BAuthor%5D&cauthor=true&cauthor_uid=18778327), et al. (2008) Polymorphism of HLA-DRB1, -DQB1 and -DPB1 genes in Bai ethnic group in southwestern China. Tissue Antigens 72:474–477.

[Huang HS](http://www.ncbi.nlm.nih.gov/pubmed?term=Huang HS%5BAuthor%5D&cauthor=true&cauthor_uid=8770634), [Peng JT](http://www.ncbi.nlm.nih.gov/pubmed?term=Peng JT%5BAuthor%5D&cauthor=true&cauthor_uid=8770634), [She JY](http://www.ncbi.nlm.nih.gov/pubmed?term=She JY%5BAuthor%5D&cauthor=true&cauthor_uid=8770634), [Zhang LP](http://www.ncbi.nlm.nih.gov/pubmed?term=Zhang LP%5BAuthor%5D&cauthor=true&cauthor_uid=8770634), [Chao CC](http://www.ncbi.nlm.nih.gov/pubmed?term=Chao CC%5BAuthor%5D&cauthor=true&cauthor_uid=8770634), et al. (1995) HLA-encoded susceptibility to insulin-dependent diabetes mellitus is determined by DR and DQ genes as well as their linkage disequilibria in a Chinese population. Hum Immunol 44:210–219.

Huang LJ, Liu W, Cui Y. (2004) Relationship between HLA-DQA1, -DQB1 alleles polymorphism and coronary heart disease (in Chinese). **Zhonghua Jian Yan Yi Xue Za Zhi** 27:677.

Huang YK, Wang MF, Hao P, Yang W, Qi Q, et al. (2007) The polymorphism of HLA-DRB1 and -DQB1 genes in Kunming Bai nationality children (in Chinese). Zhongguo Mian Yi Xue Za Zhi 25:833–834.

[Hwang SJ](http://www.ncbi.nlm.nih.gov/pubmed?term=Hwang SJ%5BAuthor%5D&cauthor=true&cauthor_uid=11972876), [Chu CW](http://www.ncbi.nlm.nih.gov/pubmed?term=Chu CW%5BAuthor%5D&cauthor=true&cauthor_uid=11972876), [Huang DF](http://www.ncbi.nlm.nih.gov/pubmed?term=Huang DF%5BAuthor%5D&cauthor=true&cauthor_uid=11972876), [Lan KH](http://www.ncbi.nlm.nih.gov/pubmed?term=Lan KH%5BAuthor%5D&cauthor=true&cauthor_uid=11972876), [Chang FY](http://www.ncbi.nlm.nih.gov/pubmed?term=Chang FY%5BAuthor%5D&cauthor=true&cauthor_uid=11972876), et al. (2002) Genetic predispositions for the presence of cryoglobuiinemia and serum autoantibodies in Chinese patients with chronic hepatitis C. Tissue Antigens 59:31–37.

Ji HZ, Duan SY, Hu LP, Gu P, Gong WZ, et al. (2005) HLA-DQA1-DQB1 linked gene haplotype in elderly patients with Impaired glucose tolerance (in Chinese). Zhongguo Lao Nian Xue Za Zhi 25:508–510.

[Jiang YG](http://www.ncbi.nlm.nih.gov/pubmed?term=Jiang YG%5BAuthor%5D&cauthor=true&cauthor_uid=14562382), [Wang YM](http://www.ncbi.nlm.nih.gov/pubmed?term=Wang YM%5BAuthor%5D&cauthor=true&cauthor_uid=14562382), [Liu TH](http://www.ncbi.nlm.nih.gov/pubmed?term=Liu TH%5BAuthor%5D&cauthor=true&cauthor_uid=14562382), [Liu J](http://www.ncbi.nlm.nih.gov/pubmed?term=Liu J%5BAuthor%5D&cauthor=true&cauthor_uid=14562382). (2003) Association between HLA class Ⅱgene and susceptibility or resistance to chronic hepatitis B. World J Gastroenterol 9:2221-2225.

Jin Y, Fu HJ, Weng MW. (2003) Association of HLA-DRB1, DQA1, DQB1 in Patients with Bullous Pemphigoid (in Chinese). Zhonghua Pi Fu Ke Za Zhi 36:368–371.

[Jin P](http://www.ncbi.nlm.nih.gov/pubmed?term=Jin P%5BAuthor%5D&cauthor=true&cauthor_uid=21470281), [Huang G](http://www.ncbi.nlm.nih.gov/pubmed?term=Huang G%5BAuthor%5D&cauthor=true&cauthor_uid=21470281), [Lin J](http://www.ncbi.nlm.nih.gov/pubmed?term=Lin J%5BAuthor%5D&cauthor=true&cauthor_uid=21470281), [Yang L](http://www.ncbi.nlm.nih.gov/pubmed?term=Yang L%5BAuthor%5D&cauthor=true&cauthor_uid=21470281), [Xiang B](http://www.ncbi.nlm.nih.gov/pubmed?term=Xiang B%5BAuthor%5D&cauthor=true&cauthor_uid=21470281), et al. (2011) High titre of antiglutamic acid decarboxylase autoantibody is a strong predictor of the development of thyroid autoimmunity in patients with type 1 diabetes and latent autoimmune diabetes in adults. Clin Endocrinol 74:587–592.

[Kelly MA](http://www.ncbi.nlm.nih.gov/pubmed?term=Kelly MA%5BAuthor%5D&cauthor=true&cauthor_uid=7759307), [Zhang Y](http://www.ncbi.nlm.nih.gov/pubmed?term=Zhang Y%5BAuthor%5D&cauthor=true&cauthor_uid=7759307), [Penny MA](http://www.ncbi.nlm.nih.gov/pubmed?term=Penny MA%5BAuthor%5D&cauthor=true&cauthor_uid=7759307), Jacobs KH, Cavan DA, et al. (1995) Genetic susceptibility to multiple sclerosis in a Shanghai Chinese population. The role of the HLA Class II genes. Hum Immunol 42:203-208.

Li LZ, Sun Li, Han JW, Zhao WC, Hai R, et al. (2012) The interrelated research between HLA-Cw*0602，-DQB1 and Psoriasis Vulgaris in Mongolian nationals in Inner Mongolia (in Chinese).Zhongguo Pi Fu Xing Bing Xue Za Zhi 26(:283-286.

[Li PK](http://www.ncbi.nlm.nih.gov/pubmed?term=Li PK%5BAuthor%5D&cauthor=true&cauthor_uid=7773959), [Poon AS](http://www.ncbi.nlm.nih.gov/pubmed?term=Poon AS%5BAuthor%5D&cauthor=true&cauthor_uid=7773959), [Tsao SY](http://www.ncbi.nlm.nih.gov/pubmed?term=Tsao SY%5BAuthor%5D&cauthor=true&cauthor_uid=7773959), [Ho S](http://www.ncbi.nlm.nih.gov/pubmed?term=Ho S%5BAuthor%5D&cauthor=true&cauthor_uid=7773959), [Tam JS](http://www.ncbi.nlm.nih.gov/pubmed?term=Tam JS%5BAuthor%5D&cauthor=true&cauthor_uid=7773959), et al. (1995) No association between HLA-DQ and -DR genotypes with nasopharyngeal carcinoma in southern Chinese. Cancer Genet Cytogenet 81:42–45.

Li NZ, Ying ZJ, Pan SQ, Huang JF, Huang SX, et al. (1996) Study on the Association between HLA-DQA1/DQB1 Genes and genetic susceptibility of Graves disease in Han Chinese (in Chinese). Zhonghua Yi Xue Za Zhi 76:55–56.

Li SZ, Jiang YJ, Xie BW, Zhang XH, Du X, et al. (2011) Correlation between polymorphisms of DRB1，-DQA1, and -DQB1 alleles and susceptibility to pulmonary tuberculosis in Tibetan population of China (in Chinese). Di San Juan Yi Da Xue Xue Bao 33:1254-1257.

Li SN, Wang B, Wu YR, Tang WR, Tang L, et al. (2008) Polymorphism of HLA-DQB1 gene in Dong ethnic group of Guizhou Province (in Chinese). Yunnan Da Xue Xue BO 30:384–386.

Li X, Zhang XN, Zheng QP, Shao HG, Liu P, et al. (1998) PCR-PFLP Polymorphism of HLA-DQA1/DQB1 in the Tibetans of Qinghai Province (in Chinese). Yi Chuan Xue Bao 25:398–402.

Li YZ, Lu WK, Gao Y, Zhang Y, Mao TS. (1998) On the HLA-DR-DQ-gene haplotype and susceptibility of SPIDDM and FPIDDM in adults (in Chinese). Zhonghua Nei Fen Mi Dai Xie Za Zhi 14:302–304.

Liang J, Chu L, Xu Z, Zhang LF, Cao X, et al. (2008) Association between polymorphisms in HLA- II genes and multiple sclerosis in Guizhou province (in Chinese). Zhonghua Sheng Jing Ke Za Zhi 41:83-86.

Liang XH, Yu WJ, Hu RH, Wang B. (2004) Association between HLA-DRB1, -DQB1 alleles and HBV cirrhosis (in Chinese). Zhongguo Shu Xue Za Zhi 17:352-353.

Lin SC, Sun A. (1990) HLA-DR and DQ antigens in Chinese patients with oral lichen planus. J Oral Pathol Med 19:298–300.

Lin J, Deng CS, Zhu YQ, Xiong P, Wang YP. (2002) The genetic susceptibility of HLA-DRB1, -DQB1 alleles in esophageal squamous cell carcinoma (in Chinese). Zhonghua Xiao Hua Za Zhi 22:726–729.

Lin J, Deng CS, Xiong P, Wang YP. (2001) Study on the genetic susceptibility of HLA-DRB1,-DQB1 alleles in colorectal neoplasm of hubei Han Chinese (in Chinese) .Zhonghua Jian Yan Yi Xue Za Zhi 24:156-157.

[Lin JH](http://www.ncbi.nlm.nih.gov/pubmed?term=Lin JH%5BAuthor%5D&cauthor=true&cauthor_uid=12878363), [Liu ZH](http://www.ncbi.nlm.nih.gov/pubmed?term=Liu ZH%5BAuthor%5D&cauthor=true&cauthor_uid=12878363), [Lv FJ](http://www.ncbi.nlm.nih.gov/pubmed?term=Lv FJ%5BAuthor%5D&cauthor=true&cauthor_uid=12878363), [Fu YG](http://www.ncbi.nlm.nih.gov/pubmed?term=Fu YG%5BAuthor%5D&cauthor=true&cauthor_uid=12878363), [Fan XL](http://www.ncbi.nlm.nih.gov/pubmed?term=Fan XL%5BAuthor%5D&cauthor=true&cauthor_uid=12878363), et al. (2003) Molecular Analyses of HLA-DRB1, -DPB1, and -DQB1 in Jing Ethnic Minority of Southwest China. Hum Immunol 64:830–834.

Liu Q, Zhang MF, Qiu CC, Hu TS. (1999) Association of HLA-DQA1 and DQB1 alleles with Vogt-Koyanagi-Harada syndrome in Han Chinese Population (in Chinese). Zhonghua Yan Ke Za Zhi 35:200–215.

Liu CL, Yu YR, Liu H, Zhang XX, Zhao GZ. (2004) The associations of HLA-DQB1 gene with onset age and autoantibodies in type 1 diabetes (in Chinese). Zhonghua Yi Xue Yi Chuan Xue Za Zhi 21:368–371.

Liu W, Li WM, Sun NL. (2005) HLA-DQA1, -DQB1 Polymorphism and genetic susceptibility to idiopathic dilated cardiomyopathy in Hans of Northern China. Ann Hum Genet 69: 382-388.

[Liu Y](http://www.ncbi.nlm.nih.gov/pubmed?term=Liu Y%5BAuthor%5D&cauthor=true&cauthor_uid=16441488), [Liu Z](http://www.ncbi.nlm.nih.gov/pubmed?term=Liu Z%5BAuthor%5D&cauthor=true&cauthor_uid=16441488), [Fu Y](http://www.ncbi.nlm.nih.gov/pubmed?term=Fu Y%5BAuthor%5D&cauthor=true&cauthor_uid=16441488), [Jia Z](http://www.ncbi.nlm.nih.gov/pubmed?term=Jia Z%5BAuthor%5D&cauthor=true&cauthor_uid=16441488), [Chen S](http://www.ncbi.nlm.nih.gov/pubmed?term=Chen S%5BAuthor%5D&cauthor=true&cauthor_uid=16441488), et al. (2006) Polymorphism of HLA class II genes in Miao and Yao nationalities of Southwest China. Tissue Antigens 67(2):157–159.

Liu YZ, Yang P, Xie HS, Dang RM, Yu YS. (2011) Research of HLA-DRB1 and HLA-DQB1 gene polymorphisms on the Buyi nationality of Guizhou (in Chinese). Xian Dai Yu Fang Yi Xue Za Zhi 38 :319-320.

Long GF , A Abdi Mohamed.(1998) Association of HLA-DQB1 alleles and the susceptibility to β–thalassemia in Guangxi Chinese Zhuang nationality (in Chinese). Zhonghua Xue Ye Xue Za Zhi 19:528–530.

Long GF, Pan SL, Lin WX, Chen JC, Huang BG, et al. (2000) A study on DNA polymorphism of HLA-DQA1 and DQB1 loci in Zhuang nationality in Barma county and its association with human longevity (in Chinese). Zhonghua Lao Nian Yi Xue Za Zhi 19:289–291.

[Lu LY](http://www.ncbi.nlm.nih.gov/pubmed?term=Lu LY%5BAuthor%5D&cauthor=true&cauthor_uid=9182925), [Ding WZ](http://www.ncbi.nlm.nih.gov/pubmed?term=Ding WZ%5BAuthor%5D&cauthor=true&cauthor_uid=9182925), [Fici D](http://www.ncbi.nlm.nih.gov/pubmed?term=Fici D%5BAuthor%5D&cauthor=true&cauthor_uid=9182925), [Deulofeut R](http://www.ncbi.nlm.nih.gov/pubmed?term=Deulofeut R%5BAuthor%5D&cauthor=true&cauthor_uid=9182925), [Cheng HH](http://www.ncbi.nlm.nih.gov/pubmed?term=Cheng HH%5BAuthor%5D&cauthor=true&cauthor_uid=9182925), et al. (1997) Molecular analysis of major histocompatibility complex allelic associations with systemic lupus erythematosus in Taiwan. Arthritis Rheum 40:1138–1145.

Lu YF, Zuo YG. (2009) HLA-DQB1 gene polymorphism in patients with recurrent condyloma acuminatum (in Chinese). Zhonghua Pi Fu Ke Za Zhi 42:851–852.

[Magira EE](http://www.ncbi.nlm.nih.gov/pubmed?term=Magira EE%5BAuthor%5D&cauthor=true&cauthor_uid=12626563), [Papaioakim M](http://www.ncbi.nlm.nih.gov/pubmed?term=Papaioakim M%5BAuthor%5D&cauthor=true&cauthor_uid=12626563), [Nachamkin I](http://www.ncbi.nlm.nih.gov/pubmed?term=Nachamkin I%5BAuthor%5D&cauthor=true&cauthor_uid=12626563), [Asbury AK](http://www.ncbi.nlm.nih.gov/pubmed?term=Asbury AK%5BAuthor%5D&cauthor=true&cauthor_uid=12626563), [Li CY](http://www.ncbi.nlm.nih.gov/pubmed?term=Li CY%5BAuthor%5D&cauthor=true&cauthor_uid=12626563), et al. (2003) Differential distribution of HLA-DQ beta/DR beta epitopes in the two forms of Guillain-Barré syndrome, acute motor axonal neuropathy and acute inflammatory demyelinating polyneuropathy (AIDP): identification of DQ beta epitopes associated with susceptibility to and protection from AIDP. J Immunol 170:3074–3080.

[Mizuki M](http://www.ncbi.nlm.nih.gov/pubmed?term=Mizuki M%5BAuthor%5D&cauthor=true&cauthor_uid=9389328), [Ohno S](http://www.ncbi.nlm.nih.gov/pubmed?term=Ohno S%5BAuthor%5D&cauthor=true&cauthor_uid=9389328), [Ando H](http://www.ncbi.nlm.nih.gov/pubmed?term=Ando H%5BAuthor%5D&cauthor=true&cauthor_uid=9389328), [Sato T](http://www.ncbi.nlm.nih.gov/pubmed?term=Sato T%5BAuthor%5D&cauthor=true&cauthor_uid=9389328), [Imanishi T](http://www.ncbi.nlm.nih.gov/pubmed?term=Imanishi T%5BAuthor%5D&cauthor=true&cauthor_uid=9389328), et al. (1997) Major histocompatibility complex class II alleles in Kazak and Han populations in the Silk Route of northwestern China. Tissue Antigens 50:527–534.

Pan SL, Liu CW, Long GF, Li WX, Zhou XL, et al. (2005) Polymorphism of HLA-DQA1 and HLA-DQB1 loci of Chuang nationality longevous population in Donglan County of Guangxi (in Chinese). Zhongguo Lao Nian Xue Za Zhi 25:233–235.

[Rudwaleit M](http://www.ncbi.nlm.nih.gov/pubmed?term=Rudwaleit M%5BAuthor%5D&cauthor=true&cauthor_uid=7677450), [Gibson K](http://www.ncbi.nlm.nih.gov/pubmed?term=Gibson K%5BAuthor%5D&cauthor=true&cauthor_uid=7677450), [Wordsworth P](http://www.ncbi.nlm.nih.gov/pubmed?term=Wordsworth P%5BAuthor%5D&cauthor=true&cauthor_uid=7677450), [Pile K](http://www.ncbi.nlm.nih.gov/pubmed?term=Pile K%5BAuthor%5D&cauthor=true&cauthor_uid=7677450), [Oh V](http://www.ncbi.nlm.nih.gov/pubmed?term=Oh V%5BAuthor%5D&cauthor=true&cauthor_uid=7677450). (1995) HLA associations of systemic lupus erythematosus in Chinese from Singapore. Ann Rheum Dis 54:686-687.

Sang YM, Yan C, Zhu C, Zhu C, Ni GC. (1997) A study on dose-effect rules of the relation ship between human leukocyte antigen DQ alleles and genetic susceptibility of insulin dependent diabetes mellitus (in Chinese). Zhonghua Er Ke Za Zhi 35:304–308.

[Shaw CK](http://www.ncbi.nlm.nih.gov/pubmed?term=Shaw CK%5BAuthor%5D&cauthor=true&cauthor_uid=9458114), [Chang TK](http://www.ncbi.nlm.nih.gov/pubmed?term=Chang TK%5BAuthor%5D&cauthor=true&cauthor_uid=9458114), [Chen SN](http://www.ncbi.nlm.nih.gov/pubmed?term=Chen SN%5BAuthor%5D&cauthor=true&cauthor_uid=9458114), [Wu S](http://www.ncbi.nlm.nih.gov/pubmed?term=Wu S%5BAuthor%5D&cauthor=true&cauthor_uid=9458114). (1997) HLA polymorphism and probability of finding HLA-matched unrelated marrow donors for Chinese in Taiwan. Tissue Antigens 50: 610–619.

Shen JJ, Tan YH, Guan XL, Huang XJ, Guo YH, et al. (1997) Study on HLA-DR-DQ haplotypes in Xinjiang Uygur (in Chinese). Zhonghua Yi Xue Yi Chuan Xue Za Zhi 14:234–238.

Si TM, Shu L, Kong FH. (1998) Human leukocyte antigen association with schizophrenia] (in Chinese). Zhongguo Shen Jing Jing Shen Ji Bing Za zhi 24:81-83.

[Su X](http://www.ncbi.nlm.nih.gov/pubmed?term=Su X%5BAuthor%5D&cauthor=true&cauthor_uid=18001300), [Bi L](http://www.ncbi.nlm.nih.gov/pubmed?term=Bi L%5BAuthor%5D&cauthor=true&cauthor_uid=18001300), [Hai R](http://www.ncbi.nlm.nih.gov/pubmed?term=Hai R%5BAuthor%5D&cauthor=true&cauthor_uid=18001300), [Qimuge S](http://www.ncbi.nlm.nih.gov/pubmed?term=Qimuge S%5BAuthor%5D&cauthor=true&cauthor_uid=18001300), [Ying M](http://www.ncbi.nlm.nih.gov/pubmed?term=Ying M%5BAuthor%5D&cauthor=true&cauthor_uid=18001300), et al. (2007) HLA-DPB1, -DRB1, and -DQB1 polymorphism defined in Ewenki ethnic minority of China Inner Mongolia Autonomous Region. Int J Immunogenet 34:435–440.

Sun YP, Gao XJ, Qiu JN, Li HZ, Tan YH,et al.(1992) HLA Class II genetic variability in North and South groups of China (in Chinese). Zhongguo Mian Yi Xue Za Zhi 8:283–286.

[Sun A](http://www.ncbi.nlm.nih.gov/pubmed?term=Sun A%5BAuthor%5D&cauthor=true&cauthor_uid=8445544), [Lin SC](http://www.ncbi.nlm.nih.gov/pubmed?term=Lin SC%5BAuthor%5D&cauthor=true&cauthor_uid=8445544), [Chu CT](http://www.ncbi.nlm.nih.gov/pubmed?term=Chu CT%5BAuthor%5D&cauthor=true&cauthor_uid=8445544), [Chiang CP](http://www.ncbi.nlm.nih.gov/pubmed?term=Chiang CP%5BAuthor%5D&cauthor=true&cauthor_uid=8445544). (1993) HLA-DR and DQ antigens in Chinese patients with Behcet's disease. J Oral Pathol Med 22:60–63.

Sun XF, Li Q, Sun YP, Huang YY, Liao BP, et al. (1997) The polymorphism of HLA class II alleles in Guangdong Han nationality (in Chinese). Zhonghua Yi Xue Yi Chuan Xue Za Zhi 14:270–273.

[Sun A](http://www.ncbi.nlm.nih.gov/pubmed?term=Sun A%5BAuthor%5D&cauthor=true&cauthor_uid=11488417), [Hsieh RP](http://www.ncbi.nlm.nih.gov/pubmed?term=Hsieh RP%5BAuthor%5D&cauthor=true&cauthor_uid=11488417), [Chu CT](http://www.ncbi.nlm.nih.gov/pubmed?term=Chu CT%5BAuthor%5D&cauthor=true&cauthor_uid=11488417), [Wang JT](http://www.ncbi.nlm.nih.gov/pubmed?term=Wang JT%5BAuthor%5D&cauthor=true&cauthor_uid=11488417), [Liu BY](http://www.ncbi.nlm.nih.gov/pubmed?term=Liu BY%5BAuthor%5D&cauthor=true&cauthor_uid=11488417), et al. (2001) Some specific human leukocyte antigen (HLA)-DR/DQ haplotypes are more important than individual HLA-DR and -DQ phenotypes for the development of mucocutaneous type of Behcet’s disease and for disease shift from recurrent aphthous stomatitis to mucocutaneous type of Behcet’s disease. J Oral Pathol Med 30:402–407.

Tang GY, Xu GQ, Zhang GL,Zheng SQ, Ji CH. (1999) Analysis of HLA antigens in patients with oral lichen planus (in Chinese).Shi Yang Kou Qiang Yi Xue Za Zhi 1999;15(3):175-176.

[Trachtenberg E](http://www.ncbi.nlm.nih.gov/pubmed?term=Trachtenberg E%5BAuthor%5D&cauthor=true&cauthor_uid=17900288), [Vinson M](http://www.ncbi.nlm.nih.gov/pubmed?term=Vinson M%5BAuthor%5D&cauthor=true&cauthor_uid=17900288), [Hayes E](http://www.ncbi.nlm.nih.gov/pubmed?term=Hayes E%5BAuthor%5D&cauthor=true&cauthor_uid=17900288), [Hsu YM](http://www.ncbi.nlm.nih.gov/pubmed?term=Hsu YM%5BAuthor%5D&cauthor=true&cauthor_uid=17900288), [Houtchens K](http://www.ncbi.nlm.nih.gov/pubmed?term=Houtchens K%5BAuthor%5D&cauthor=true&cauthor_uid=17900288), et al. (2007) HLA class I (A, B, C) and class II (DRB1, DQA1, DQB1, DPB1) alleles and haplotypes in the Han from southern China. Tissue Antigens 70:455–463.

Tsai SC, Sheen MC, Chen BH. (2011) Association between HLA-DQA1, HLA-DQB1 and oral cancer. Kaohsiung J Med Sci 27:441-445.

[Waine GJ](http://www.ncbi.nlm.nih.gov/pubmed?term=Waine GJ%5BAuthor%5D&cauthor=true&cauthor_uid=9602373), [Ross AG](http://www.ncbi.nlm.nih.gov/pubmed?term=Ross AG%5BAuthor%5D&cauthor=true&cauthor_uid=9602373), [Williams GM](http://www.ncbi.nlm.nih.gov/pubmed?term=Williams GM%5BAuthor%5D&cauthor=true&cauthor_uid=9602373), [Sleigh AC](http://www.ncbi.nlm.nih.gov/pubmed?term=Sleigh AC%5BAuthor%5D&cauthor=true&cauthor_uid=9602373), [McManus DP](http://www.ncbi.nlm.nih.gov/pubmed?term=McManus DP%5BAuthor%5D&cauthor=true&cauthor_uid=9602373). (1998) HLA class II antigens are associated with resistance or susceptibility to hepatosplenic disease in a Chinese population infected with Schistosoma japonicum. Int J Parasitol 28:537–542.

Wang QF, Liao YH, Gong FL Mao HY, Zhang JZ. (2000) HLA-DQB1 gene polymorphism in patients with dilated cardiomyopathy (in Chinese). Lin Chuang Xin Xue Guan Bing Za Zhi 16:115–117.

Wang WP, Wang JZ, Li CY, Guo L, Liu RC, et al. (2001) A study of the association between acute axonal neuropathy and HLA (in Chinese). Zhongguo Shen Jing Jing Shen Ji Bing 27:250-253.

[Wang M](http://www.ncbi.nlm.nih.gov/pubmed?term=Wang M%5BAuthor%5D&cauthor=true&cauthor_uid=14990915), [Xing ZM](http://www.ncbi.nlm.nih.gov/pubmed?term=Xing ZM%5BAuthor%5D&cauthor=true&cauthor_uid=14990915), [Yu DL](http://www.ncbi.nlm.nih.gov/pubmed?term=Yu DL%5BAuthor%5D&cauthor=true&cauthor_uid=14990915), [Yan Z](http://www.ncbi.nlm.nih.gov/pubmed?term=Yan Z%5BAuthor%5D&cauthor=true&cauthor_uid=14990915), [Yu LS](http://www.ncbi.nlm.nih.gov/pubmed?term=Yu LS%5BAuthor%5D&cauthor=true&cauthor_uid=14990915). (2004) Association between HLA class II locus and the susceptibility to Artemisia pollen–induced allergic rhinitis in Chinese population. [Otolaryngol Head Neck Surg](http://www.ncbi.nlm.nih.gov/pubmed/14990915#%23) 130:192-196.

[Wang JF](http://www.ncbi.nlm.nih.gov/pubmed?term=Wang JF%5BAuthor%5D&cauthor=true&cauthor_uid=16904993), [Zhang D](http://www.ncbi.nlm.nih.gov/pubmed?term=Zhang D%5BAuthor%5D&cauthor=true&cauthor_uid=16904993), [Zhao JZ](http://www.ncbi.nlm.nih.gov/pubmed?term=Zhao JZ%5BAuthor%5D&cauthor=true&cauthor_uid=16904993), [Jia BX](http://www.ncbi.nlm.nih.gov/pubmed?term=Jia BX%5BAuthor%5D&cauthor=true&cauthor_uid=16904993), [Bi RM](http://www.ncbi.nlm.nih.gov/pubmed?term=Bi RM%5BAuthor%5D&cauthor=true&cauthor_uid=16904993). (2006) A study on the relationship between HLA-DR, DQ antigen, and intracranial aneurysm in the Han nationality. Surg Neurol 66:S25-29.

[Wang JP](http://www.ncbi.nlm.nih.gov/pubmed?term=Wang JP%5BAuthor%5D&cauthor=true&cauthor_uid=17919266), [Zhou ZG](http://www.ncbi.nlm.nih.gov/pubmed?term=Zhou ZG%5BAuthor%5D&cauthor=true&cauthor_uid=17919266), [Lin J](http://www.ncbi.nlm.nih.gov/pubmed?term=Lin J%5BAuthor%5D&cauthor=true&cauthor_uid=17919266), [Huang G](http://www.ncbi.nlm.nih.gov/pubmed?term=Huang G%5BAuthor%5D&cauthor=true&cauthor_uid=17919266), [Zhang C](http://www.ncbi.nlm.nih.gov/pubmed?term=Zhang C%5BAuthor%5D&cauthor=true&cauthor_uid=17919266), et al. (2007) Islet autoantibodies are associated with HLA-DQ genotypes in Han Chinese patients with type 1 diabetes and their relatives. Tissue Antigens 70:369–375.

Wang JP, Zhang C, Lin J, Yuan Y, Zhou HF, et al. (2007) Relationship between autoantibodies and HLA-DQ genotypes in patients with type1 diabetes mellitus (in Chinese). Zhonghua Yi Xue Za Zhi 87:2380–2384.

Wang J, Zhao YM, Wang Y, Xiao Y, Wang YK, et al. (2007) Association of HLA Class and alleles with generalized vitiligo in Chinese Hans in north China (in Chinese). Zhonghua Yi Xue Yi Chuan Xue Za Zhi 24:221–223.

Wang Q, Yao YF, Shi L, Shi L, Sun H, et al. (2009) The Polymorphism of HLA-DRB1 and -DQB1 genes in Bai ethnic group in Yunnan Province (in Chinese). Zhongguo Mian Yi Xue Za Zhi 25:1088–1091.

Wang X, Liu YC, He P, Wu F, Wang P, et al. (2010) Association between HLA-DQBl gene polymorphisms and tuberculosis in Xingjiang Uygur Population (in Chinese). Xi Bao Yu Fen Zi Mian Yi Xue Za Zhi 26:494–496.

Wen GS, Huang YK, Hao P, Qi Q, Li HL, et al. (2004) Analysis of HLA–DRB1, DQB1 allele polymorphism in the Kunming Yi nationality population (in Chinese). Zhonghua Yi Xue Yi Chuan Xue Za Zhi 21:522–523.

Wen GS, Huang YK, Hao P, Li HL, Qi Q, et al. (2005) Immunogenetic analysis of human leukocyte antigen DRB1,DQB1 locus among Han ethnic children with Helicobacter pylori infection in Kunming (in Chinese). Zhonghua Liu Xing Bing Xue Za Zhi 26:286–289.

Wu XM, Xiao LH, Wang CD, Zhang KN, Qu XH, et al. (2011) Study of association between human leucocyte antigen-DQB1 allele gene polymorphism and multiple sclerosis in Han population from southern area of China (in Chinese). Lin Chuang Shen Jing Bing Xue Za Zhi 24:11-13.

[Xiao FL](http://www.ncbi.nlm.nih.gov/pubmed?term=Xiao FL%5BAuthor%5D&cauthor=true&cauthor_uid=16231148), [Zhou FS](http://www.ncbi.nlm.nih.gov/pubmed?term=Zhou FS%5BAuthor%5D&cauthor=true&cauthor_uid=16231148), [Liu JB](http://www.ncbi.nlm.nih.gov/pubmed?term=Liu JB%5BAuthor%5D&cauthor=true&cauthor_uid=16231148), [Yan KL](http://www.ncbi.nlm.nih.gov/pubmed?term=Yan KL%5BAuthor%5D&cauthor=true&cauthor_uid=16231148), [Cui Y](http://www.ncbi.nlm.nih.gov/pubmed?term=Cui Y%5BAuthor%5D&cauthor=true&cauthor_uid=16231148), et al. (2005) Association of HLA-DQA1 and DQB1 alleles with alopecia areata in Chinese Hans. Arch Dermatol Res 297:201–209.

Xie ZF, Fan LA, Chen GM, Chen N, Yang JQ, et al. (2000) HLA-DQA1, DQB1 alleles associated with primary IgA nephropathy (in Chinese). Zhonghua Shen Zang Bing Za Zhi 16:395–396.

Xing WJ, Zhang SL, Ke BS, Huang XY, Jiang ZS, et al. (2001) HLA-DPB1, DQB1 genes and their correlation with autoantibodies in type 1 diabetes mellitus (in Chinese). Zhonghua Nei Fen Mi Dai Xie Za Zhi 17:338–340.

Xu LD, Fan L, Chen N, Yang JQ, Yao FJ. (1999) Study on HLA-DQB1 allele polymorphism in primary IgA nephropathy (in Chinese). Mian Yi Xue Za Zhi 15:253–255.

Xu JR, Yang Y, Zhang DT, Gao LY, Wang YJ. (2009) Analysis on polymorphisms of HLA-D QB1 gene in Ningxia Hui polulation with essential hypertension] (in Chinese). Jilin Da Xue Xue Bao (Medicine Edition) 35:1103-1106.

Xu XP, Wang CY, Cao JF, Liu RY, Wu YY, et al. (1992) Study on the DNA typing of HLA class Ⅱ genes in Chinese Buyi nationality (in Chinese). Zhonghua Wei Sheng Wu Xue He Mian Yi Xue Za Zhi 12:285-289.

Xu XP, Wang CY, Cao JF, Liu RY, Wu YY, et al. (1993) Study on the polymorphism of serological typing on HLA class Ⅰ, Ⅱ antigens in Buyi nationality] (in Chinese). Zhonghua Wei Sheng Wu Xue He Mian Yi Xue Za Zhi 13:226-228.

[Yang KL](http://www.ncbi.nlm.nih.gov/pubmed?term=Yang KL%5BAuthor%5D&cauthor=true&cauthor_uid=19480851), [Chen SP](http://www.ncbi.nlm.nih.gov/pubmed?term=Chen SP%5BAuthor%5D&cauthor=true&cauthor_uid=19480851), [Shyr MH](http://www.ncbi.nlm.nih.gov/pubmed?term=Shyr MH%5BAuthor%5D&cauthor=true&cauthor_uid=19480851), [Lin PY](http://www.ncbi.nlm.nih.gov/pubmed?term=Lin PY%5BAuthor%5D&cauthor=true&cauthor_uid=19480851). (2009) High-resolution human leukocyte antigen (HLA) haplotypes and linkage disequilibrium of HLA-B and -C and HLA-DRB1 and -DQB1 alleles in a Taiwanese population. Hum Immunol 70:269-276.

Yang W, Wang MF, Huang YK, Hao P, Qi Q, et al. (2007) Immunogenetic analysis of HLA-DR/DQB1 in Kunming Bai nationality children with H. pylori infection (in Chinese). Lin Chuang Er Ke Za Zhi 25: 679–682.

Yu GL, Sun YP, Xu LM, Gao FY, Li GQ, et al. (1995) A study on HLA–DR, DQ, DP DNA typing in Shenyang Han populations (in Chinese). Zhongguo Mian Yi Xue Za Zhi 11:142–145.

[Yu HX](http://www.ncbi.nlm.nih.gov/pubmed?term=Yu HX%5BAuthor%5D&cauthor=true&cauthor_uid=10527399), [Thai AC](http://www.ncbi.nlm.nih.gov/pubmed?term=Thai AC%5BAuthor%5D&cauthor=true&cauthor_uid=10527399), [Chan SH](http://www.ncbi.nlm.nih.gov/pubmed?term=Chan SH%5BAuthor%5D&cauthor=true&cauthor_uid=10527399). (1999)HLA Microsatellite associations with insulin-dependent diabetes mellitus in Singaporean Chinese. Hum Immunol 60:894-900.

Yu RB, Hong X, Ding WL, Tan YF, Wu GL. (2006) Polymorphism of the HLA-DQAl and -DQBl genes of Han population in Jiangsu Province, China. Zhonghua Yi Xue Za Zhi 19:1930–1933.

[Yu RB](http://www.ncbi.nlm.nih.gov/pubmed?term=Yu RB%5BAuthor%5D&cauthor=true&cauthor_uid=18028350), [Hong X](http://www.ncbi.nlm.nih.gov/pubmed?term=Hong X%5BAuthor%5D&cauthor=true&cauthor_uid=18028350), [Ding WL](http://www.ncbi.nlm.nih.gov/pubmed?term=Ding WL%5BAuthor%5D&cauthor=true&cauthor_uid=18028350), [Tan YF](http://www.ncbi.nlm.nih.gov/pubmed?term=Tan YF%5BAuthor%5D&cauthor=true&cauthor_uid=18028350), [Zhang YX](http://www.ncbi.nlm.nih.gov/pubmed?term=Zhang YX%5BAuthor%5D&cauthor=true&cauthor_uid=18028350), et al. (2008) The association between the genetic polymorphism of HLA-DQA1, DQB1, and DRB1 and serum alanine aminotransferase levels in chronic hepatitis C in the Chinese population. J Gastroen Hepatol 23:1394-1402.

Zha XS, Xuan GW, Chen H, Fan LQ, Chen DC. (2004) Study on the relationship between HLA –DQ DNA typing and susceptibility of Systemic Lupus Erythematosus in Hans Nationality Chinese of Guangdong Province (in Chinese). Zhongguo Pi Fu Xing Bing Xue Za Zhi 18:86–87,100.

[Zhai L](http://www.ncbi.nlm.nih.gov/pubmed?term=Zhai L%5BAuthor%5D&cauthor=true&cauthor_uid=17305280), [Sun Y](http://www.ncbi.nlm.nih.gov/pubmed?term=Sun Y%5BAuthor%5D&cauthor=true&cauthor_uid=17305280), [Tang L](http://www.ncbi.nlm.nih.gov/pubmed?term=Tang L%5BAuthor%5D&cauthor=true&cauthor_uid=17305280), [Liu H](http://www.ncbi.nlm.nih.gov/pubmed?term=Liu H%5BAuthor%5D&cauthor=true&cauthor_uid=17305280). (2007) Polymorphism between loci for human leukocyte antigens DR and DQ in patients with nasal polyps. Ann Otol Rhinol Laryngol 116:66-68.

Zhang HY, Wang BY, Sun JH, et al. (2001) The study on the association of HLA-DQB1 alleles and type1 diabetes in childhood (in Chinese). Zhongguo Tang Niao Bing Za Zhi 9(5):263-265.

Zhang QR, Song FJ, Li HG, Geng L, Xiao Y, et al. (1994) [Association between HLA and condyloma] (in Chinese). Zhonghua Yi Xue Yi Chuan Xue Za Zhi 11: 362-364.

Zhang XN, Pazilaiti, Shao HG, Zhu DL, Geng ZC. (1996) [HLA-DQA1 and -DQB1 genotyping by PCR–RFLP in Xinjiang Han Chinese] (in Chinese). Zhongguo Mian Yi Xue Za Zhi 12:89–92.

Zhang W, Chen N, Dong DH, Fei HM, Jiang WH. (1996) Study on HLA-DQA1，DQB1 genes associated with idiopathic membranous nephropathy (in Chinese). Zhonghua Wei Sheng Wu Xue He Mian Yi Xue Za Zhi 16:435–437.

Zhang WB, Shen CW, Cai TD, Yang YQ, Yao FJ, et al. (1997) A primary study of the association of human leucocyte antigen and osteosarcoma (in Chinese). Zhonghua Zhong Liu Za Zhi 19 :309–312.

Zhang XN, Liu YG, Pazilaiti, Li D, Li X, et al. (1998) Polymorohism of HLA-DQA1,-DQB1 Genes in Uygur and Kazak ethnic groups and comprehensive analysis of 25 related populations (in Chinese).Yi Chuan Xue Bao 25:193–198.

Zhang Q, Xiong P, Wei H, Fang JM, Gong FL, et al. (2000) The relationship between HLA-DRB1 and -DQB1 and the susceptibility for autoimmune thyroid disease (in Chinese). Zhonghua Er Ke Za Zhi 38:361–364.

Zhong XM, Xu CD, Xi RP, Chen SN, Xu CD, et al. (2005) A study on the association of HLA-DQB1 allele with duodenal ulcer and Helicobacter pylori infection in children (in Chinese). Lin Chuang Er Ke Za Zhi 23:692–694.

[Zhou GP](http://www.ncbi.nlm.nih.gov/pubmed?term=Zhou GP%5BAuthor%5D&cauthor=true&cauthor_uid=8018489), [Guo YQ](http://www.ncbi.nlm.nih.gov/pubmed?term=Guo YQ%5BAuthor%5D&cauthor=true&cauthor_uid=8018489), [Ji YH](http://www.ncbi.nlm.nih.gov/pubmed?term=Ji YH%5BAuthor%5D&cauthor=true&cauthor_uid=8018489), [Zhang GL](http://www.ncbi.nlm.nih.gov/pubmed?term=Zhang GL%5BAuthor%5D&cauthor=true&cauthor_uid=8018489). (1994) Major histocompatibility complex class II antigens in steroid-sensitive nephrotic syndrome in Chinese children. Pediatr Nephrol 8:140–141.

Zhou SH, Lin L, Jin PY, Ye SZ. (2002) Study on association between HLA class Ⅱ genes and Pemphigus Erythematosus in Han Chinese (in Chinese). Zhongguo Pi Fu Xing Bing Xue Za Zhi 16:145-147.

Zhou SH, Gao Y, Yang L, Wang XM, Zhang YJ, et al. (2005) Study on the association between HLA-DRB1, DQB1 genes and atopic dermatitis in Han Chinese (in Chinese). Zhongguo Pi Fu Xing Bing Xue Za Zhi 19:267–268,285.

Zhou SH, Wang XM, Han ZD, Gao Y, Zhang YJ, et al. (2007) Study on the association between HLA-DRBl genes and Bullous Pemphigoid in Shandong Han Chinese (in Chinese). Zhongguo Pi Fu Xing Bing Xue Za Zhi 21:72–74.

Zhu WG, Jin SZ, Bao ZQ, Zou HY, Li Z, et al. (2009) Polymorphism and linkage disequilibrium analysis of HLA-A, -C, -B, -DRB1 and -DQB1 genes at high-resolution level in Chinese Han population from Guangdong province (in Chinese). Zhongguo Shu Xue Za Zhi 22:987–993.
